# Responses of A549 human lung epithelial cells to cristobalite and α‐quartz exposures assessed by toxicoproteomics and gene expression analysis

**DOI:** 10.1002/jat.3420

**Published:** 2016-12-05

**Authors:** Ngoc Q. Vuong, Patrick Goegan, Francesco De Rose, Dalibor Breznan, Errol M. Thomson, Julie S. O'Brien, Subramanian Karthikeyan, Andrew Williams, Renaud Vincent, Premkumari Kumarathasan

**Affiliations:** ^1^ Inhalation Toxicology LaboratoryEnvironmental Health Science and Research Bureau, Health CanadaOttawaONK1A 0K9Canada; ^2^ Analytical Biochemistry and Proteomics LaboratoryEnvironmental Health Science and Research Bureau, Health CanadaOttawaONK1A 0K9Canada; ^3^ Biostatistics Section, Population Studies DivisionEnvironmental Health Science and Research Bureau, Health CanadaOttawaONK1A 0K9Canada; ^4^ Department of Biochemistry, Faculty of ScienceUniversity of OttawaOttawaONK1H 8M5Canada

**Keywords:** cristobalite, α‐quartz, cytotoxicity, A549 cells, 2D‐GE: two‐dimensional gel electrophoresis, RT‐PCR, silica, proteomics, genomics

## Abstract

In this study, we used cytotoxicity assays, proteomic and gene expression analyses to examine the difference in response of A549 cells to two silica particles that differ in physical properties, namely cristobalite (CR) and α‐quartz (Min‐U‐Sil 5, MI). Cytotoxicity assays such as lactate dehydrogenase release, 5‐bromo‐2′‐deoxyuridine incorporation and cellular ATP showed that both silica particles could cause cell death, decreased cell proliferation and metabolism in the A549 human lung epithelial cells. While cytotoxicity assays revealed little difference between CR and MI exposures, proteomic and gene expression analyses unveiled both similar and unique molecular changes in A549 cells. For instance, two‐dimensional gel electrophoresis data indicated that the expression of proteins in the cell death (e.g., ALDH1A1, HTRA2 and PRDX6) and cell proliferation (e.g., FSCN1, HNRNPAB and PGK1) pathways were significantly different between the two silica particles. Reverse transcription–polymerase chain reaction data provided additional evidence supporting the proteomic findings. Preliminary assessment of the physical differences between CR and MI suggested that the extent of surface interaction between particles and cells could explain some of the observed biological effects. However, the differential dose–response curves for some other genes and proteins suggest that other physical attributes of particulate matter can also contribute to particulate matter‐related cellular toxicity. Our results demonstrated that toxicoproteomic and gene expression analyses are sensitive in distinguishing subtle toxicity differences associated with silica particles of varying physical properties compared to traditional cytotoxicity endpoints. Copyright © 2016 Her Majesty the Queen in Right of Canada. *Journal of Applied Toxicology* published by John Wiley & Sons, Ltd.

## Abstract 2

In this study, we used cytotoxicity assays, proteomic and gene expression analyses to examine the difference in response of A549 cells to two silica particles that differ in physical properties, namely cristobalite (CR) and α‐quartz (Min‐U‐Sil 5, MI). Cytotoxicity assays such as lactate dehydrogenase release, 5‐bromo‐2'‐deoxyuridine incorporation and cellular ATP showed that both silica particles could cause cell death, decreased cell proliferation and metabolism in the A549 human lung epithelial cells. While cytotoxicity assays revealed little difference between CR and MI exposures, proteomic and gene expression analyses unveiled both similar and unique molecular changes in A549 cells.

## Introduction

Inhalation of silica has been reported to cause pulmonary fibrosis or silicosis, a condition where scar tissue is formed in the lung (AGN, 1930; Belt, 1930; Cassel *et al*., 2008). It is known that exposure to silica can cause inflammatory response (Cassel *et al*., 2008; Dostert *et al*., 2008; Hornung *et al*., 2008; Peeters *et al*., 2013) and cell death (Cassel *et al*., 2008; Chao *et al*., 2001; Iyer *et al*., 1996; Joshi & Knecht, 2013) in the cells of the respiratory tract. Recent studies demonstrated the NALP3 inflammasome is a protein complex (composed of NLRP3, ASC and caspase‐1) that is essential for the inflammatory response (Cassel *et al*., 2008; Dostert *et al*., 2008; Hornung *et al*., 2008; Peeters *et al*., 2013) and the development of silicosis (Cassel *et al*., 2008) from silica exposure. However, there are discrepancies in the molecular mechanisms reported for the effect of silica on the cells. For example, data from Cassel *et al*. (2008) and Dostert *et al*. (2008) demonstrated that the inflammatory response depends on the production of reactive oxygen species (ROS), whereas data from Hornung *et al*. (2008) suggested that the production of ROS is not necessary. These discrepancies may arise from the difference in materials and/or the methods used in these studies. The silica particles used in these three studies were 1.5 μm amorphous silicon dioxide (Dostert *et al*., 2008), Min‐U‐Sil 5 (MI; quartz, 5 μm top size) (Cassel *et al*., 2008) and Min‐U‐Sil 15 (quartz, 15 μm top size) (Hornung *et al*., 2008).

Given that silica or silicon dioxide can form various physical structures such as amorphous (non‐crystalline) and crystalline (e.g., quartz, cristobalite [CR] and tridymite), it is possible that exposure to different forms of silica may lead to different health impacts. To date, most toxicological studies as mentioned above have focused on quartz, and very limited data have been reported on the toxicity of other forms of silica. There is a wide range of current industrial products/applications for different forms of silica, which include clay, ceramics, road building, sand blasting, pet litter, electronic devices and cosmetic products (IARC, 1997, 2012). Thus, there is a need to distinguish the difference in toxicity of different forms of silica, if there is any, to inform the workers or consumers about the hazard of the relevant materials. Thereby, it is fundamental to assess whether different forms of silica can trigger differential responses at the cellular level. Recently, we demonstrated that toxicoproteomics is a useful approach to identify and differentiate the mechanisms of particle toxicity of two respirable particles that are physically and chemically different such as titanium dioxide and carbon black (Vuong *et al*., 2016a). In this study, we questioned whether *in vitro* toxicoproteomics in conjunction with gene expression analysis were sensitive enough to differentiate the effect of two particles that are identical in chemical formula (silicon dioxide) but differed only in their physical properties. The results of this study demonstrated that subtle differences in cytotoxic effects of CR and MI on A549 human lung epithelial cells could be addressed through *in vitro* toxicoproteomic and gene expression analyses.

## Materials and methods

### Materials

Culture flasks (T‐25 and T‐75), 96‐well plates and plastic cell scrapers were obtained from Corning Inc. (Corning, NY, USA). Dulbecco's modified Eagle's medium and fetal bovine serum were purchased from Hyclone (Logan, UT, USA). Gentamicin, trifluoroacetic acid, α‐cyano‐4‐hydroxy‐cinnamic acid, Tris‐HCl, NaCl, Tween‐20 and Tween‐80 were obtained from Sigma‐Aldrich (Oakville, ON, Canada). Iodoacetamide, bis‐acrylamide, ammonium persulfate, glycerol, immobilized pH gradient strips, Criterion Cassette (13.3 cm × 8.7 cm W × L), Tris/glycine/sodium dodecyl sulfate buffer and BioSafeCoomassie Blue were purchased from Bio‐Rad (Mississauga, ON, Canada). Trypsin, resazurin reduction (CellTiter‐Blue®) and lactate dehydrogenase (LDH) cytotoxicity assay kits (CytoTox‐96®) were from Promega Corporation (Madison, WI, USA), ATP assay kit (ViaLight™ Plus) was from Lonza Corporation (Rockland, ME, USA) and 5‐bromo‐2′‐deoxyuridine (BrdU) cell proliferation enzyme‐linked immunosorbent assay (chemiluminescent) assay kit was obtained from Roche Diagnostics (Laval, QC, Canada). All water used was deionized/demineralized (>16 MΩ resistivity).

### Particle preparation

CR (SRM‐1879a) was obtained from NIST (Gaithersburg, MD, USA) and MI was a generous gift from the US Silica Co. (Berkeley Springs, WV, USA). Both silica particles were subjected to three successive washes with methanol followed by 1× phosphate‐buffered saline to remove possible soluble metals and organic contaminants before use in the experiments (Vincent *et al*., 1997). Particles were resuspended at 10 mg ml^–1^ in particle buffer (0.19% NaCl and 25 μg ml^–1^ Tween‐80) (Nadeau *et al*., 1996), vortexed (30 s), sonicated (20 min on ice), homogenized with a Dounce homogenizer (25 strokes) and then heated (56 °C, 1 h). The particles were stored at –40 °C until use.

### Scanning electron microscopy

The size and morphology of CR and MI samples were characterized by scanning electron microscopy (SEM). Images were collected on a JSM‐7500F FESEM (JEOL, Peabody, MA, USA) instrument equipped with a field emission gun under the following parameters: beam acceleration voltage, 2 kV; working distance, between 7 and 9 mm; imaging mode, lower secondary electron image. Magnification and sizing bar are as indicated in the figure captions for each individual image. Samples were prepared by dropping a small amount of powder on to an aluminum stage painted with carbon paint (electron microscope sciences). The paint was allowed to dry for 20 min, and the excess powder was then removed by blowing the surface with compressed, dry air. Particle size distributions were calculated from SEM images using the software program ImageJ. The measurements reported are the average of at least 100 random particles. For all particles that were not spherical, the longest axis was measured and reported.

### Cell culture and particle exposure

The A549 cell line (American Type Culture Collection, Manassas, VA, USA; CCL‐185; human, epithelial, lung carcinoma) was subcultured in Dulbecco's modified Eagle's medium supplemented with 50 μg ml^–1^ gentamicin and 10% fetal bovine serum. The cells were maintained in T‐75 flasks in a humidified atmosphere containing 5% CO_2_ and 95% air at 37 °C. For experiments, the cells were seeded at 1.5 × 10^6^ cells/T‐25 flask (for gene expression analysis and proteomics) or 2.0 × 10^4^ cells per well (96‐well plate for cytotoxicity assays) and incubated for 24 h, resulting in approximately 75% confluence before dosing with particles. The final volume of culture medium was 5 ml (T‐25), 15 ml (T‐75) or 200 μl per well (96‐well plate). Solutions of particles were prepared by thawing the frozen stocks, sonicating on ice (20 min) then diluting in the culture medium to generate dosing concentrations of 0, 60, 140 and 200 μg cm^–2^. The cells were exposed to the particles by replacing the existing culture medium with the particle‐containing medium, and the flasks/plates were returned to the incubator for a 24 h exposure to particles. To harvest the exposed cells, the medium in each flask was removed and the cells were detached from the flasks using a plastic scraper. The cell suspension was collected in cell culture medium and centrifuged at 350 *g* for 5 min, and the supernatant was discarded. The cell pellet was then washed twice with phosphate‐buffered saline. The final cell pellet was aspirated dry and stored frozen at –80 °C until further use. The integrated cytotoxicity bioassay, which combined endpoints of cell viability (resazurin reduction assay), cellular membrane integrity (intracellular LDH release) and energy metabolism (ATP assay), was conducted in a 96‐well plate as described previously (Kumarathasan *et al*., 2015). The cell proliferation (BrdU incorporation) assay was performed in a separate 96‐well plate.

### Protein extraction and two‐dimensional gel electrophoresis

Total protein from the A549 cells (control and particle‐exposed) was extracted and examined by two‐dimensional gel electrophoresis (2D‐GE) as previously described (Vuong *et al*., 2016a,b). Following electrophoresis, the gel was washed for 30 min in water, stained in BioSafeCoomassie Blue (Bio‐Rad) overnight (16–20 h), destained twice in water (20 min) and then imaged with a standard scanner. To overcome the typical warping and distortion issues from gel to gel particularly near the extremities of the pH range and the molecular weight, a common area across all experimental gels that clearly shows the protein spots was selected to assess the proteome differences among the treatments, where proteins in the window of pH 5.1–7.8 and 100–20 kDa were analyzed (Vuong *et al*., 2016a,b). A total of 543 well‐resolved protein spots in this common area were compared across all experimental gels, and the identities of 333 of these protein spots were determined via matrix‐assisted laser desorption/ionization time‐of‐flight/time‐of‐flight mass spectrometry (MALDI‐TOF‐TOF‐MS; Vuong *et al*., 2016a,b). The protein spots within the gels were matched and quantified with PDQuestTM Advance V8.0.1 (Bio‐Rad), where spot volume was quantified using the available “Local regression model (LOESS)” algorithm in PDQuest. The reported spot volume for each protein was used to compare its level of expression across the treatments.

### Gene expression analysis

Total RNA from cell pellets of different treatments was extracted, quantified and prepared for gene expression analysis by reverse transcription–polymerase chain reaction (RT‐PCR) as described elsewhere (Thomson *et al*., 2015). Four house‐keeping genes (RPL32, ACTB, HPRT1 and YWHAZ) were assessed as potential reference genes for normalization purposes according to stability across treatments (Chen *et al*., 2011), and RPL32 was found as the least affected gene across all treatments (data not shown).

### Statistical analyses

Two‐way analysis of variance (ANOVA) was performed on 2D‐GE (*n* = 3), RT‐PCR (*n* = 3) and cytotoxicity (LDH, BrdU, ATP and CTB; *n* = 4) data with treatment and dose as factors, using R (R Core Team, 2013). When the assumptions of equal variance and normal distribution were not met, the data were rank‐transformed. Holm–Sidak was the *post‐hoc* method used for all pairwise comparisons. A data point was considered as having a significant effect if *P* < 0.05. If the *Treatment* × *Dose* interaction was significant for a protein spot or gene, its change in expression for a given treatment and dose that was found significant by Holm–Sidak analysis was reported as it is (as seen in Supporting information Tables S1 and S3). The same applied for those proteins or genes that were found to have significant *Treatment* and *Dose* main effects. If a protein was found to have a significant *Treatment* main effect, fold‐changes (FCs) were estimated using least square mean (Goodnight & Harvey, 1978; Searle *et al*., 1980). In the case where the *Dose* main effect was significant, the average FC estimate was reported for each significant dose group.

Pearson correlation analysis was conducted on 2D‐GE (*n* = 3), RT‐PCR (*n* = 3) and cytotoxicity (LDH, BrdU, ATP and CTB; *n* = 4) data using R (R Core Team, 2013). The FC in all data sets was transformed to Log2(FC) to ensure that all data are linear and continuous. Correlation between the responses of A549 cells to the doses of silica particles was conducted on two dose metrics, namely mass (e.g., of 0, 60, 140 and 200 μg cm^–2^ for both CR and MI) and surface area (SA; e.g., 0, 32, 74 and 106 mm^2^ cm^–2^ for CR and 0, 54, 127 and 181 mm^2^ cm^–2^ for MI) (Table 1). Correlations were calculated based on pooled data from both CR and MI for each protein spot, gene or assay. Venn diagrams used to assess the similarities and/or differences in responses in A549 cells following particle exposures were generated via VENNY (Oliveros, 2015).

**Table 1:** Physical characteristics (density and diameter) of the silica particles, and the doses expressed in mass or SA in which the A549 cells were exposed

	Cristobalite				Min‐U‐Sil 5			
Density	2.27 g cm^–3^				2.65 g cm^–3^			
Median diameter	5.0 μm				2.5 μm			
Calculated SA	0.53 mm^2^ μg^–1^				0.91 mm^2^ μg^–1^			
Mass dose (μg cm^–2^)	0	60	140	200	0	60	140	200
SA dose (mm^2^ cm^–2^)*	0	32	74	106	0	54	127	181

SA, surface area.

* Values were calculated based on the assumption that the individual particles have a spherical shape.

#### Bioinformatic analysis (pathway analysis)

2D‐GE data showed that multiple protein spots with the same protein ID may have *P* < 0.05, which suggests different isoforms of the same protein were significantly altered, and thus rigorous assessment may be required for proper interpretation of biological implication if the directions of change of these isoforms are different. However, our data showed that the expression of all significant protein spots with the same ID aligned in the same direction. Therefore, we simply chose the protein with the greatest FC (either increase or decrease) to conduct pathway analysis. Furthermore, protein spots that were deemed as small peptides/fragments (based on molecular weight and unique peptide sequences) of their native proteins were excluded from pathway analysis, unless functional data can be found for such peptides based on PubMed (http://www.ncbi.nlm.nih.gov/pubmed) and UniProt (www.uniprot.org) searches. For pathway analysis, an FC cut‐off of ±1.10 and ±1.50 were applied on top of the significantly changed (*P* < 0.05) protein spots and genes, respectively, to filter out nuanced changes in expressions that may not contribute to any biological impact. Pathway analysis was conducted using Ingenuity Pathway Analysis (www.ingenuity.com). When conducting pathway analysis, only the pathways that were influenced by more than five significant proteins or genes in any particle treatment group were flagged. This arbitrary cut‐off was set to identify the more probable cellular pathways that could be affected in A549 cells following particle exposure.

## Results

### Physical properties of the silica particles

**Figure 1:**
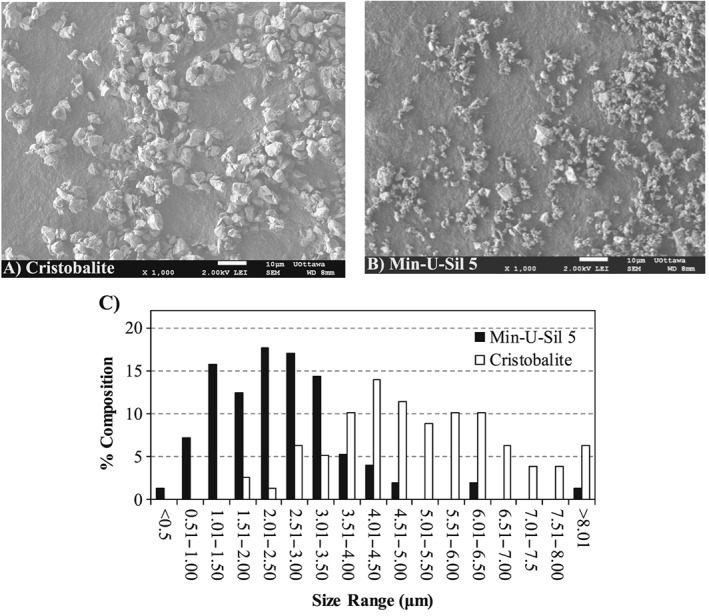
Size and shape of the particles in cristobalite (A) and Min‐U‐Sil 5 (B) samples observed by scanning electron microscopy. (C) Distribution of the particle size in the silica samples, where the median particle size for cristobalite is about 5.0 μm and Min‐U‐Sil 5 is about 2.5 μm.

The SEM image in Fig. 1(B) showed that MI particles cover a broad range of sizes. The particles are irregularly shaped, possessing both sharp edges and flat, stepped terraces, consistent with a crystalline sample. There is a lack of porosity observed on the surface of the particles, which is consistent with a highly crystalline solid sample that has been ground from a larger, non‐porous sample. CR particles have a similar appearance to that of MI, but the CR particles are more uniform in size (Fig. 1A). CR particles were distributed over a size range of 1.5 to >8 μm and MI were distributed over a broader size range of <0.5 to >8 μm (Fig. 1C). There is an overlap in particle size distribution between CR and MI from 1.5 to >8 μm, and MI has more particles in smaller sizes. The median particle sizes for CR and MI are 5.0 and 2.5 μm, respectively. The A549 cell monolayers were exposed to CR and MI at particle mass doses of 0, 60, 140 and 200 μg cm^–2^, and the corresponding particle SA doses for CR were 0, 32, 74 and 106 mm^2^ cm^–2^, and for MI were 0, 54, 127 and 181 mm^2^ cm^–2^ (Table 1).

### Cytotoxicity assays

**Figure 2:**
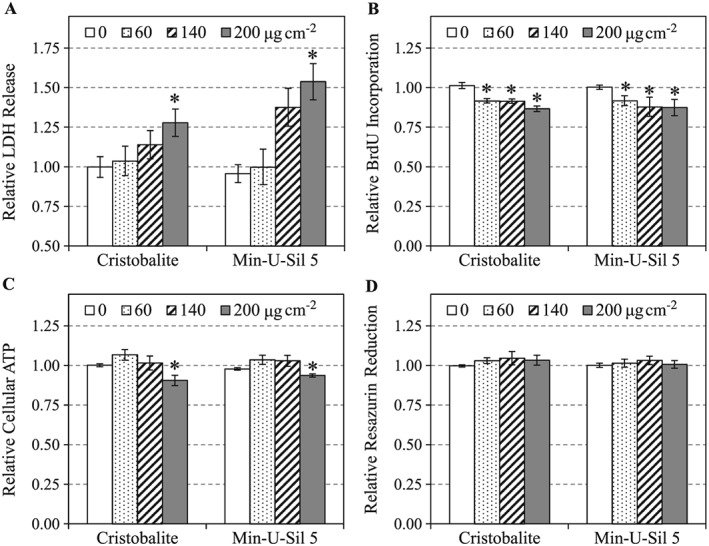
Cytotoxicities of cristobalite and Min‐U‐Sil 5 in A549 cells after 24 h of exposure were assessed by LDH release (A), BrdU incorporation (B), cellular ATP (C) and resazurin reduction (D) assays. Data are expressed as mean fold effect ± standard error, relative to control (0 μg cm^–2^), *n* = 4. Two‐way ANOVA was used to determine significant effects of the particles, where Holm–Sidak was the *post‐hoc* method used for all pairwise comparison procedures. *Significant change (*P* < 0.05) compared to control (0 μg cm^–2^). BrdU, 5‐bromo‐2′‐deoxyuridine; LDH, lactate dehydrogenase.

The LDH assay in Fig. 2(A), which assessed the integrity of the cell membrane, showed that both silica particles can cause a significant leakage of LDH from A549 cells at the highest dose, but there was no significant difference in cytotoxicity caused by CR and MI (two‐way ANOVA: *Dose* main effect, *P* < 0.05; Holm–Sidak: 200 vs 0 μg cm^–2^, *P* < 0.05). Cellular proliferation measured by BrdU incorporation (Fig. 2B) revealed that both silica particles decreased the mitotic activity of A549 cells to similar levels at all doses (two‐way ANOVA: *Dose* main effect, *P* < 0.05; Holm–Sidak: 60, 140 and 200 vs 0 μg cm^–2^, *P* < 0.05). Both particles also significantly decreased cellular ATP at the highest dose to a similar level (Fig. 2C) (two‐way ANOVA: *Dose* main effect, *P* < 0.05; Holm–Sidak: 200 vs 0 μg cm^–2^, *P* < 0.05). There was no significant change in resazurin reduction in A549 cells exposed to any dose of CR and MI (Fig. 2D).

### Particulate matter‐induced changes in the proteome of A549 cells examined by two‐dimensional gel electrophoresis

**Figure 3:**
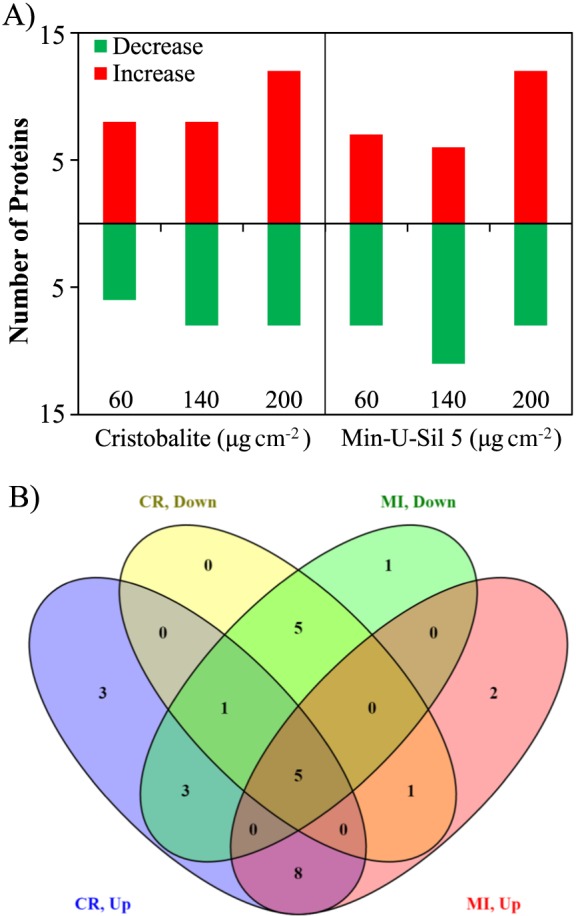
Changes in two‐dimensional gel electrophoresis protein spots (*P* < 0.05; two‐way ANOVA) associated with *in vitro* exposure of A549 cells to silica particles. (A) Bar graph shows the number of proteins that increased or decreased in expression following particle treatments at doses 60, 140 and 200 μg cm^–2^. (B) Venn diagram shows the number of protein spots that exhibited particle‐specific and non‐specific changes. CR, cristobalite; MI, Min‐U‐Sil 5.

Changes in the proteome of A549 cells after 24 h of exposure to CR and MI particles were assessed via 2D‐GE as described previously (Vuong *et al*., 2016a,b). The results presented in Supporting information Table S1 revealed that the expressions of 49 protein spots were significantly affected by both CR and MI (two‐way ANOVA: *Treatment* × *Dose* interaction, *Treatment* or *Dose* main effects, *P* < 0.05). Of the significant 49 protein spots, 30 of them passed the Holm–Sidak *post‐hoc* test and their identities have also been determined by MALDI‐TOF‐TOF‐MS. 2D‐GE data showed that particle dose‐related changes were observed in half (15) of the protein spots (two‐way ANOVA: *Dose* main effect, *P* < 0.05), 14 of which are unique proteins. Particle‐specific changes were observed in nine protein spots (two‐way ANOVA: *Treatment* main effect, *P* < 0.05), all of which were unique proteins, while six protein spots exhibited *Treatment* × *Dose* interaction (two‐way ANOVA: *Treatment* × *Dose* interaction, *P* < 0.05), all six of these being unique proteins. It was noticed that greater change in the number of protein spots were observed for both particles at the higher doses (Fig. 3A). The Venn diagram in Fig. 3(B) provides an overview of the unique and similar changes in the proteome of A549 cells following CR and MI exposures. For pathway analysis, FC cut‐off was set at ±1.10.

**Figure 4:**
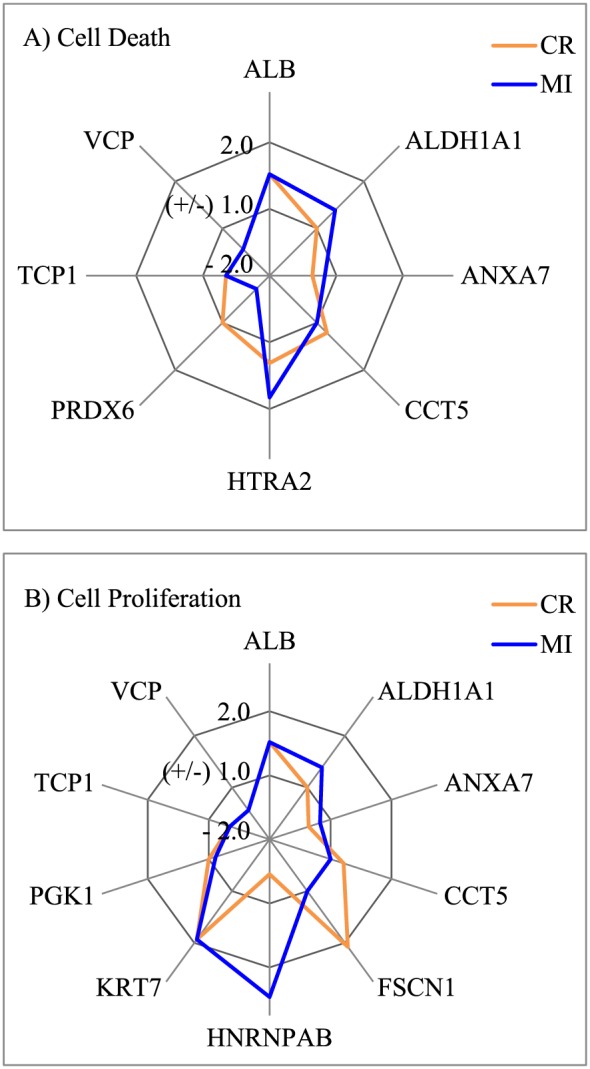
Characteristic changes in the expression of proteins involved in the cell death (A) and cell proliferation (B) pathways in A549 cells induced by CR and MI exposures at 140 μg cm^–2^. *Y*‐axis shows the fold‐change in expression relative to the control (0 μg cm^–2^). CR, cristobalite; MI, Min‐U‐Sil 5.

Pathway analysis revealed that all the proteins affected by the two silica particles were known to be associated with cell death (necrosis and/or apoptosis), proliferation, inflammation, homeostasis and cell movement pathways (Table 2). It was noticeable that CR and MI induced distinct patterns of protein expression in these pathways. For example, the spider charts in Fig. 4 demonstrated that CR and MI induced distinguishable characteristic changes in those proteins involved in the cell death and cell proliferation pathways in A549 cells.

**Table 2:** Biological functions that were estimated to be affected by the particles based on the proteins that were significantly affected using ingenuity pathway analysis. Values in the table indicate the number of significant proteins affected by the treatment. Only the pathways that were influenced by more than five proteins in any particle treatment group were shown

	Cristobalite (μg cm^–2^)			Min‐U‐Sil 5 (μg cm^–2^)		
Biological function	60	140	200	60	140	200
Mitotic activity (proliferation or cell cycle)		8	5		8	5
Cell death (apoptosis or necrosis)	6	6	8	7	7	8
Inflammatory response		5			6	
Cell movement		5		6	5	
Protein metabolism			6			5
Cellular homeostasis			6			6

### Gene expression changes in A549 cells exposed to silica particles

**Figure 5:**
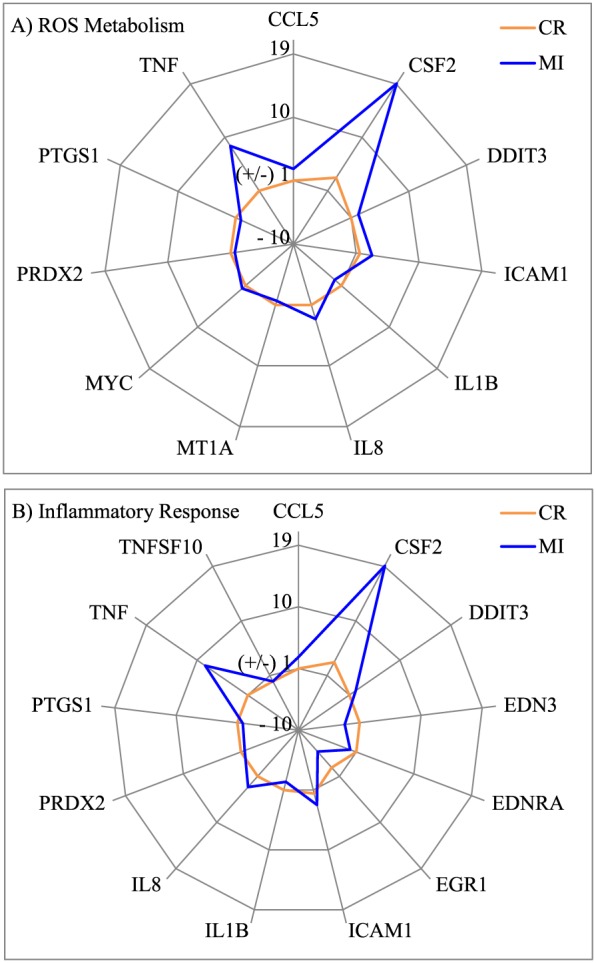
Signature changes in the expression of genes involved in the ROS metabolism (A) and inflammatory response (B) pathways in A549 cells induced by CR and MI exposures at 140 μg cm^–2^. *Y*‐axis shows the fold‐change in expression relative to the control (0 μg cm^–2^). CR, cristobalite; MI, Min‐U‐Sil 5; ROS, reactive oxygen species.

To understand better the reactivity of A549 cells to the silica particles, RT‐PCR was chosen as a gene expression analysis method to assess how CR and MI affected the expression of genes in the exposed cells. For this purpose, a panel of 89 genes was selected for gene expression analysis (Supporting information Table S2). Some of these selected genes were known to be affected by silica particles in the literature (Sellamuthu *et al*., 2011), some were genes upstream and/or downstream of those proteins in the identified pathways based on the proteomic changes in the present study, and some were selected from unrelated pathways. Of the genes selected, 37 were found to exhibit altered expression following particle exposures (Supporting information Table S3). Only two genes (CDKN1A and TNFSF10) were commonly affected by both CR and MI (two‐way ANOVA: *Dose* main effect, *P* < 0.05). The remaining genes were differentially affected by the two particles, where 12 showed *Treatment* × *Dose* interaction (two‐way ANOVA: *Treatment* × *Dose* interaction, *P* < 0.05) and 23 exhibited *Treatment* main effect (two‐way ANOVA: *Treatment* main effect, *P* < 0.05). Interestingly, the lowest dose of MI (mass: 60 μg cm^–2^) was capable of influencing a greater change in the expressions of most genes than the highest dose of CR (mass: 200 μg cm^–2^) (Supporting information Table S3). Only those genes with FC greater than ±1.50 in any treatment were used for pathway analysis. Ingenuity pathway analysis based on all the genes that were identified as significantly altered based on two‐way ANOVA (Supporting information Table S3) revealed that most of the affected genes are involved in the cell death, mitosis, homeostasis, ROS metabolism and inflammatory response pathways (Table 3). For example, the signature effects of CR and MI on those genes involved in the ROS metabolism and inflammatory response pathways in A549 cells can be clearly differentiated in the spider charts in Fig. 5.

**Table 3:** Biological functions indicated by ingenuity pathway analysis that were likely impacted by the particles based on the genes that were significantly affected. Values in the table indicate the number of genes that were significantly affected by the treatment. Only the pathways that were influenced by more than five genes in any particle treatment group were shown

	Cristobalite (μg cm^–2^)			Min‐U‐Sil 5 (μg cm^–2^)		
Biological function	60	140	200	60	140	200
Mitotic activity			7	10	16	16
Cell death (apoptosis and necrosis)			7	9	16	16
Inflammatory response			7	10	12	12
Cell movement			6	5	12	10
Lipid metabolism			6	7	12	12
Carbohydrate metabolism			6	6	12	12
ROS metabolism				7	7	9
Protein metabolism			5			7
Fibrosis				5	7	
Cell differentiation					14	9
Molecular transport					11	12
Filopodia formation						5
Hormone metabolism						5

ROS, reactive oxygen species.

### Pearson correlation

**Figure 6:**
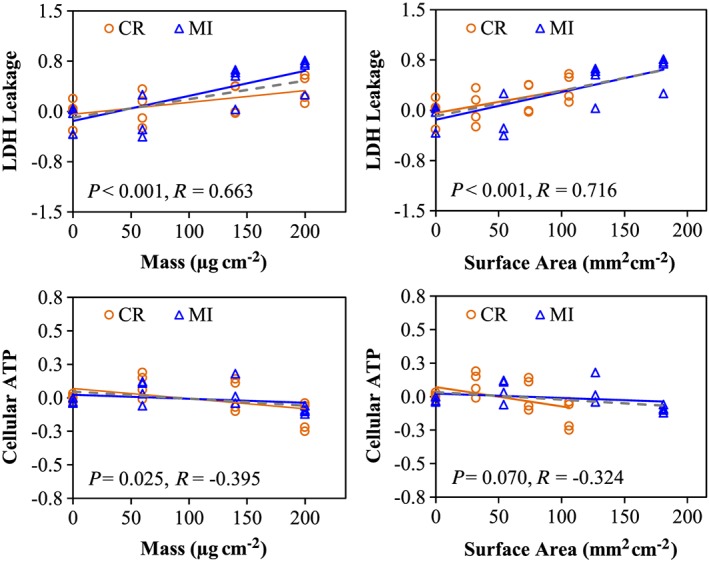
Cytotoxicity endpoints associated with mass or surface area dose metrics. Pearson correlation was conducted to assess the linear association between the responses (in Log_2_(fold‐effect)) and the doses (in mass or surface area), where *R* corresponds to the correlation coefficient and *P* < 0.05 indicates a significant correlation. Orange and blue lines correspond to the dose–response curves for CR and MI exposures, respectively, whereas the grey‐dashed line indicates the dose–response curve, when both particles are considered together. CR, cristobalite; LDH, lactate dehydrogenase; MI, Min‐U‐Sil 5. [Colour figure can be viewed at wileyonlinelibrary.com]

**Figure 7:**
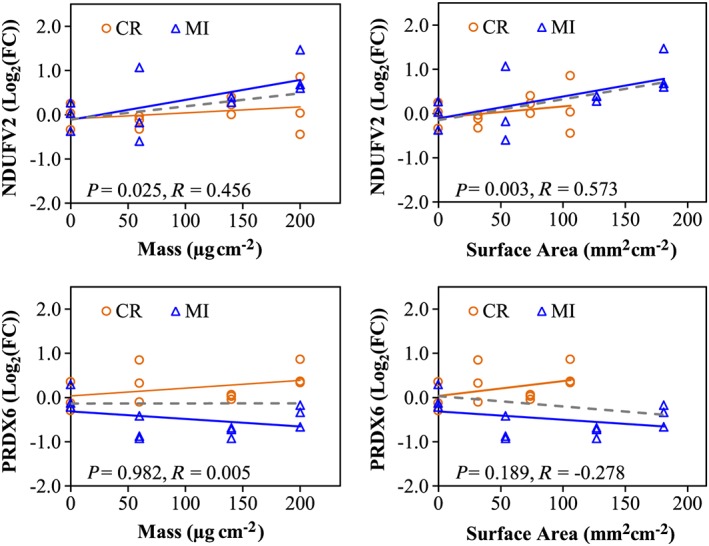
Dose–response curves for proteins based on mass or surface area. *R* corresponds to the correlation coefficient and *P* < 0.05 indicates a significant correlation based on Pearson correlation analyses. Orange and blue lines correspond to the dose–response curves for CR and MI exposures, respectively, whereas the grey‐dashed line indicates the dose–response curve, when both particles are visualized together. CR, cristobalite; FC, fold‐change; MI, Min‐U‐Sil 5. [Colour figure can be viewed at wileyonlinelibrary.com]

**Figure 8:**
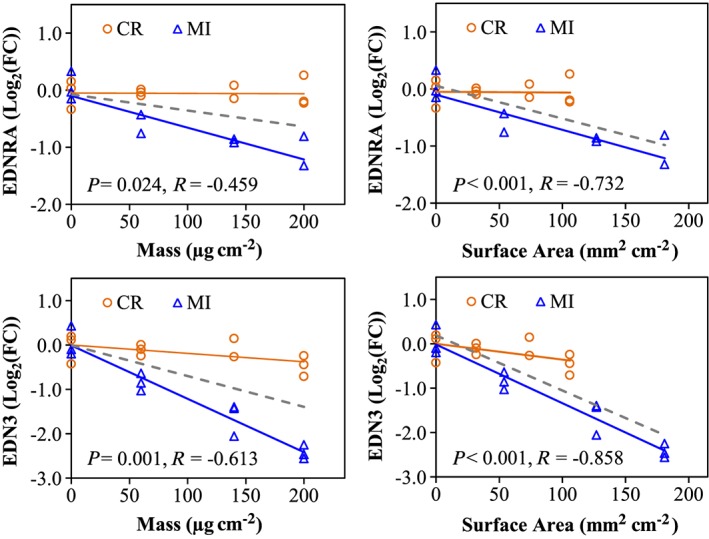
Dose–response curves for EDNRA and EDN3 genes based on mass or surface area. *R* corresponds to the correlation coefficient and *P* < 0.05 indicates significant correlation based on Pearson correlation. Orange and blue lines correspond to the dose–response curves for CR and MI exposures, respectively, whereas the grey‐dashed line indicates the dose–response curve, when both particles are considered together. CR, cristobalite; FC, fold‐change; MI, Min‐U‐Sil 5. [Colour figure can be viewed at wileyonlinelibrary.com]

It was mentioned earlier that the median particle size of CR (~5.0 μm) is larger than MI particles (~2.5 μm). Thus, a potential relationship between particle size and their differential response profiles in A549 cells was examined using Pearson correlation analysis. Pearson correlation analysis conducted on cytotoxicity assays (Supporting information Table S4) indicated that the level of cellular ATP in A549 cells correlated well with mass, whereas LDH leakage associated better with SA, while similar correlation coefficients were found for both mass and SA for BrdU incorporation. No correlation was found for either mass or SA with the pattern of cellular resazurin reduction. The dose–response plots in Fig. 6 are representative visual demonstrations of two cytotoxicity assays, namely LDH leakage and cellular ATP, which showed the dose–response curves of CR and MI exposures are similar. Pearson correlation conducted on 2D‐GE data identified 96 protein spots correlated significantly with either mass or SA (Supporting information Table S5), but stronger correlation was found with SA (81 protein spots, *P* < 0.05) than mass (46 protein spots, *P* < 0.05). Interestingly, the dose–response curves for a number of protein spots were different for the CR and MI treatments regardless if the protein spot is significantly correlated (e.g., NDUFV2 or ssp5006 in Fig. 7) or not correlated (e.g., PRDX6 or ssp7002 in Fig. 7). Similarly, Pearson correlation on gene expression in A549 cells following particle exposures showed that the dose–responses of 52 from the 88 genes examined were significantly correlated with SA and mass (Supporting information Table S6). Similar to the proteomic result, the expression of most genes associated stronger with SA than mass, and the dose–response curves of CR and MI were different for the majority of the correlated genes (Fig. 8).

## Discussion

Silica or silicon dioxide can adopt various physical structures such as amorphous (non‐crystalline) or crystalline (e.g., quartz, CR and tridymite). However, most toxicological studies thus far have focused mainly on one form of silica, which is α‐quartz, and very limited data have been reported on the toxicity of other forms of silica. The exposure regulatory limits for all forms of silica have been based on quartz. Yet, physical properties of particles can modify their toxicity characteristics. In this study, we used *in vitro* toxicoproteomic and gene expression analysis approaches to assess the difference between two silica particles, namely CR and α‐quartz (MI). The main physical differences between these two silica particles are their crystalline structures, densities and sizes (Table 1).

In this work, the A549 cells were exposed to CR and MI at relatively low toxic levels (Figs 2 and 3). Only the highest dose (200 μg cm^–2^) of CR and MI appeared to affect the cellular membrane integrity and a loss of energy content in A549 cells, potentially leading to cell death. These silica particles also decreased the mitotic activity of the cells, at all doses (Fig. 2B). These cytotoxicity assays were not able to distinguish the toxicity differences due to CR and MI exposures, except for the LDH release assay in Fig. 2(A) that suggested a possible difference in potencies (MI > CR). However, this difference did not reach statistical significance.

Toxicity changes due to these particulate matter (PM**)** exposures were also analyzed using toxicoproteomic and gene expression analysis strategies. The goal of this study was not to obtain exhaustive proteomic information, but rather to obtain some information on PM exposure‐related changes at the molecular level that can be amenable to traditional toxicity testing methods. This is a proof‐of‐principle study to test the influence of PM's physical characteristics on their toxicity properties.

Proteomic analysis was thus conducted using less costly 2D‐GE separation of proteins, with analysis for PM exposure‐related changes captured for proteins in the pH range 5.1–7.8, stained only by Coomassie Blue. Nevertheless, we observed statistically significant PM exposure‐related changes, following this protocol. For instance, CR and MI exposures led to significant (*P* < 0.05) changes in 49 protein spots based on two‐way ANOVA, where the identities of 30 of these protein spots were achieved using MALDI‐TOF‐TOF‐MS (Supporting information Table S1). Such mild changes in the cell proteome were not surprising because the exposure conditions were only moderately cytotoxic to A549 cells as seen by the cytotoxicity assay results.

In this study, we only considered the proteomic and gene expression changes that were significantly different based on the two‐way ANOVA results as differential effects associated with particle toxicity. For example, the results shown in Supporting information Table S1 indicated that there was a significant *Treatment* main effect in the expression of AUH (SSP5205) due to particle exposures. This means on average, CR treatments decreased its expression by –1.22 and MI treatments increased its expression by 1.14. It is important to understand that such FCs were relative to the control (0 μg cm^–2^). More importantly, it must also be understood that the net difference in the expression of AUH was 36% (from –1.22 to 1.14) between CR and MI exposures. Thus, such difference cannot be ignored, particularly when the *P* value is reasonably small (two‐way ANOVA: *Treatment* main effect, *P* = 0.027), and the goal of this study is to identify differential responses of A549 cells to CR and MI. When choosing an appropriate FC cut‐off value for pathway analysis, we considered that if a cut‐off were set at ±1.50, it would remove differential effects ranging from 50 to 98% (e.g., FC from 1.49 to –1.01 and FC from 1.49 to –1.49). A cut‐off at ±1.25 will filter out differential effects ranging from 25 to 48% (e.g., FC from 1.24 to –1.01 and FC from 1.24 to –1.24). A cut‐off at ±1.10 can remove all differential effects below 10% (e.g., FC from 1.09 to –1.01) and up to 18% (e.g., FC from 1.09 to –1.09). We believe that ±1.10 FC cut‐off for the significant protein spots (*P* < 0.05) and ±1.50 FC cut‐off for significant genes (*P* < 0.05) were sufficient to remove nuanced changes in expression that may not contribute to any biological impact (see Supporting information Tables S1 and S3).

When conducting pathway analysis, we chose to evaluate the effects of the particles at a subtoxic level (i.e., 140 μg cm^–2^) and yielded maximum responses from mostly “living” cells. Our results revealed that both silica particles could significantly alter the expressions of proteins that are known to be involved in cell death (necrosis and/or apoptosis), proliferation, inflammation, homeostasis, cell cycle and cell movement pathways (Table 2). Meanwhile, the cytotoxicity assay results (Fig. 2) indicated that CR and MI can cause damage to the cell membrane (an indicator of cell death), decreased BrdU incorporation (an indicator of decreased cell proliferation) and lowered cellular ATP level (an indicator of energy content) in A549 cells, which were consistent with the observed proteomic results. In addition, these findings were supported by a large body of evidence in the literature that silica particles (mostly α‐quartz in the form of Min‐U‐Sil) can induce cell death (Cassel *et al*., 2008; Chao *et al*., 2001; Iyer *et al*., 1996; Joshi & Knecht, 2013) and inflammatory response (Cassel *et al*., 2008; Dostert *et al*., 2008; Hornung *et al*., 2008; Peeters *et al*., 2013). These cellular responses were reported in respiratory tract macrophages and epithelial cells.

The spider chart in Fig. 4(A) demonstrates that more than half of the proteins (e.g., ALDH1A1, ANXA7, CCT5, HTRA2 and PRDX6) involved in the cell death pathway in A549 cells are differently altered in A549 cells due to CR and MI exposures. The GTPase calcium‐dependent phospholipid‐binding protein ANXA7 can act as an anti‐apoptotic protein (Huang *et al*., 2014, 2015; Liu *et al*., 2016; Torosyan *et al*., 2009), whereas HTRA2 (or OMI) is a pro‐apoptotic serine peptidase that is known to cleave inhibitors of apoptosis to facilitate the activation caspase 3 (Cory & Adams, 2002; Sutton *et al*., 2003; Wang *et al*., 2013). Increased expression of the pro‐apoptotic protein HTRA2 and decreased expression of the anti‐apoptotic protein ANXA7 following particle exposures suggested that both silica particles could stimulate apoptotic cell death, which were consistent with the LDH release assay (Fig. 2A). The main difference between the two particles is that MI increased the expression of the pro‐apoptotic protein HTRA2 more than its counterpart did, while CR suppressed the expression of the anti‐apoptotic protein ANXA7 more than MI. Furthermore, only MI affected the expression of antioxidant proteins such as aldehyde dehydrogenase (ALDH1A1) and peroxiredoxin‐6 (PRDX6), which are known to protect cells from apoptotic cell death (Luo *et al*., 2012; Zha *et al*., 2015). These results suggested that the MI exposures might be triggering oxidative stress pathways as MI has been reported to cause oxidative stress and apoptosis in human bronchial epithelial cells (Antognelli *et al*., 2015).

Similarly, the signature effects of CR and MI on those proteins involved in the cell proliferation pathway in A549 cells can be readily identified based on the spider chart in Fig. 4(B). The expression of ANXA7 was inhibited by both silica particles, but a stronger inhibition was noticed with CR exposure compared to MI exposure. As ANXA7 acts as an anti‐apoptotic protein (discussed above), downregulation of this protein by both silica particles can decrease the proliferation of cells. Interestingly, all ALDH1A1 (Moreb *et al*., 2008), actin‐bundling protein FSCN1 (Kano *et al*., 2010), heterogeneous nuclear ribonucleoprotein HNRNPAB (He *et al*., 2005) and ATP‐generating glycolytic enzyme phosphoglycerate kinase‐1 PGK1 (Wang *et al*., 2010) have been reported to promote mitotic activity in cells. Yet, the expression of these proteins can be stimulated (increase in cell growth) or suppressed (decrease in cell growth) depending on the particle type in the exposure. Suppressing the expression of proliferative proteins can be a mechanism of particle toxicity in A549 cells, while increased expression of proliferative proteins is likely a survival mechanism of the exposed cells. The limited proteomic analysis conducted in this study was able to distinguish particles with different physical characteristics and provided some insights into potential pathways that maybe perturbed. Exhaustive proteomic analysis through future works can provide more detailed information on mechanistic changes associated with these particle exposures.

To understand further the particle toxicity‐related molecular mechanisms, we conducted gene expression analysis via RT‐PCR to assess how the A549 cells responded to the silica particles at the transcriptional level. Gene expression analysis indicated that the two silica particles can perturb similar transcriptional mechanisms in A549 cells following 24 h of exposure (Supporting information Table S3), but the majority of the genes altered by CR and MI were particle‐specific (two‐way ANOVA: *Treatment* main effect and *Treatment* × *Dose* interaction, *P* < 0.05). For example, the lowest dose of MI (60 μg cm^–2^) was capable of influencing a greater change in the expression of most genes than the highest dose of CR (200 μg cm^–2^), clearly indicating that MI is more potent than CR (Supporting information Table S3). Furthermore, the response of a number of genes to CR and MI exposures resulted in two different curves (representative examples can be found in Fig. 8). Thus, CR and MI caused distinguishable cytotoxic effects in A549 cells at the transcriptional level.

Pathway analysis showed that the majority of the affected genes are involved pathways such as cell death (apoptosis or necrosis), cell proliferation, inflammatory response and metabolism of ROS, lipid, protein and carbohydrate (Table 3). These results were in line with our proteomic and cytotoxicity assay results. In agreement, a recent genomic study (using microarray) on the toxicity of MI reported that the particle perturbed the expression of genes in A549 cells that govern cellular growth and proliferation, cell death, inflammatory response and cell cycle (Sellamuthu *et al*., 2011). More importantly, our gene expression analysis revealed a characteristic difference between CR and MI exposures. For example, the spider charts in Fig. 5 demonstrated that the effects of CR and MI exposure were unequivocally distinct in A549 cells. The chart showed that MI induced a significantly greater level of changes than CR in the expressions of genes involved in the ROS metabolism and inflammatory response pathways; where the effect of CR in these pathways were likely non‐significant.

A physical difference between the two silica particles that may provoke differential responses in A549 cells is the grain size of the particles, where the median size of CR (~5.0 μm) particles is two times larger than MI (~2.5 μm) (Table 1). As A549 cells were exposed to equal mass dose, MI has twice the SA available to interact with the cells as compared to CR. Hence, it is possible that the responses of A549 cells to the two silica particles could be a function of SA. Thus, all the RT‐PCR, proteomic and cytotoxicity assay data were examined by linear Pearson correlation analysis to assess whether mass or SA was a better physical attribute for these biological changes. Pearson correlation for different cytotoxicity assays indicated that certain cellular responses were better associated to mass, while others were better associated to SA (Supporting information Table S4; Fig. 6). For example, LDH leakage levels are better associated with SA than with mass, which suggested that the cell surface–PM interaction could be an important mechanism in causing cell membrane damage. On the other hand, cellular ATP levels are correlated better with mass than with SA. Pearson correlation results in Supporting information Table S5 indicated that some of the proteomic responses in A549 cells were better associated with SA (81 protein spots, *P* < 0.05) than mass (46 protein spots, *P* < 0.05). Intriguingly, the dose–response relationships for a number of other proteins were not correlated with mass or SA suggesting that these protein changes may be better described by other particle physical characteristics such as crystallinity (Table 1). Similarly, RT‐PCR data also showed that the expressions of the majority of the genes examined were significantly correlated with SA and mass, but SA generally showed a stronger correlation than mass (Supporting information Table S6). As with protein changes, there were some gene expression changes that correlated less with SA or mass and these can also perhaps be explained by other physicochemical properties of PM.

Association between physical properties of these particles and the observed proteomic and gene expression responses will need to be explored further through additional studies. In summary, this study demonstrated that toxicoproteomics and gene expression analysis are sensitive strategies to dissect cellular changes relevant to the PM's physical characteristics related to the toxicity changes that are not clearly apparent by use of traditional cytotoxicity assays. This approach can be useful in understanding toxicity mechanisms mediated by environmental air PM and engineered nanomaterials.

## Conclusion

In conclusion, *in vitro* toxicoproteomics in conjunction with gene array can provide insight into the effects of physical properties of respirable PM on biological responses of A549 human lung epithelial cells. Such high‐content *in vitro* cellular toxicity data at the molecular level can be useful in discriminating toxicity mechanisms affected by the physical nature of particles.

## Conflict of interest

The authors did not report any conflict of interest**.**

## Supporting information


**Table S1**. Two‐way ANOVA results for the A549 protein spots changed due to particle exposures (*n* = 3). The SSP number corresponds to the identifier number that PDQuest used to identify the spot based on its coordinate in the gel. The number below *Treatment* main effect (Trt), *Dose* main effect (Dose) or interaction between *Treatment and Dose* (T x D) corresponds to the *p*‐value, where the bolded number emphasized *p*‐value < 0.05. Only the protein spots identified by MALDI‐TOF‐TOF‐MS/MS are provided here (Vuong et al., 2016a;Vuong et al., 2016b). The proteins indicated in red (likely degradation product of the native protein) and fold‐change indicated in blue (cut‐off at ±1.10) were excluded from pathway analysis (see Materials and Methods).
**Table S2**. Primers used in RT‐PCR to detect expression of genes in A549 cells.
**Table S3**. Two‐way ANOVA results showing significant alteration of genes in A549 cells due to particle exposures (*n* = 3). The number below *Treatment* main effect (Trt), *Dose* main effect (Dose) or interaction between *Treatment and Dose* (T x D) corresponds to the *p*‐value, where the bolded number emphasized *p*‐value < 0.05. The fold‐change of gene indicated in blue (cut‐off at ±1.10) was excluded from pathway analysis.
**Table S4**. Pearson Correlations indicating the cytotoxic effects that significantly associated (*p* < 0.05) with the dose of exposure in A549 cells. Correlation was conducted by correlating Log_2_(fold‐effect) from each cytotoxicity assay against the doses expressed in mass and surface area (SA) metrics, where R corresponds to the correlation coefficient and *p*‐value <0.05 indicates a significant correlation. Two‐way ANOVA results were also included in this table to show the significant cytotoxicity caused by CR and MI exposures. The highlighted numbers in blue and red pointed out *p*‐value less than 0.05 based on Pearson correlation and two‐way ANOVA analyses.
**Table S5**. The protein spots that were significantly correlated between their expressions and exposure doses based on Pearson correlation. Correlation was done by correlating Log_2_(fold‐change) of each protein spot against the doses expressed in mass and surface area (SA) metrics as shown in Table 1, where R corresponds to the correlation coefficient and *p*‐value <0.05 indicates a significant correlation. The SSP number corresponds to the identifier number that PDQuest used to identify the spot based on its coordinate in the gel. #N/A indicates the protein spots whose identity is not available. Two‐way ANOVA result was also included in this table to show the protein spots that were differentially expressed in A549 cells due to CR and MI exposures. The highlighted numbers in blue and red pointed out *p*‐value less than 0.05 based on Pearson correlation and two‐way ANOVA analyses.
**Table S6**. Pearson correlation conducted to identify significant responses of genes in A549 cell to the exposure dose regardless of the type of silica particle. Correlation was done by correlating Log_2_(fold‐change) of each gene against the doses expressed in mass and surface area (SA) as shown in Table 1, where R corresponds to the correlation co‐efficient and *p*‐value <0.05 indicates a significant correlation. Two‐way ANOVA result was also included in this table to show the genes that were differentially expressed in A549 cells due to CR and MI exposures. The highlighted numbers in blue and red pointed out *p*‐value less than 0.05 based on Pearson correlation and two‐way ANOVA analyses.

Supporting info item
