# Supplementary material for: Responses of A549 human lung epithelial cells to cristobalite and α‐quartz exposures assessed by toxicoproteomics and gene expression analysis
Source: J Appl Toxicol. 2016 Dec 5;37(6):721–31. doi: 10.1002/jat.3420 (PMC5434822; doi:10.1002/jat.3420)
Supplement: Supplementary file 1 — Supporting info item [file JAT-37-721-s001.docx]

Table S1. Two-way ANOVA results for the A549 protein spots changed due to particle exposures (n = 3). The SSP number corresponds to the identifier number that PDQuest used to identify the spot based on its coordinate in the gel. The number below *Treatment* main effect (Trt), *Dose* main effect (Dose) or interaction between *Treatment and Dose* (T x D) corresponds to the p-value, where the bolded number emphasized p-value<0.05. Only the protein spots identified by MALDI-TOF-TOF-MS/MS are provided here (Vuong et al., 2016a;Vuong et al., 2016b). The proteins indicated in red (likely degradation product of the native protein) and fold-change indicated in blue (cut-off at ±1.10) were excluded from pathway analysis (see Materials and Methods).

|  |  | **^§^Cristobalite (μg/cm^2^)** | | | **^§^Min-U-Sil 5 (μg/cm^2^)** | | | **Two-way ANOVA** | | | **^†^Cristobalite (μg/cm^2^)** | | | **^†^Min-U-Sil 5 (μg/cm^2^)** | | |
| --- | --- | --- | --- | --- | --- | --- | --- | --- | --- | --- | --- | --- | --- | --- | --- | --- |
| **SSP** | **ID** | **60** | **140** | **200** | **60** | **140** | **200** | **Trt** | **Dose** | **T x D** | **60** | **140** | **200** | **60** | **140** | **200** |
| 1005 | PNMA6C | -1.03 | -1.13 | 1.01 | 7.23 | 8.06 | 7.24 | **0.042** | 0.086 | **0.043** |  |  |  | 7.23 | 8.06 | 7.24 |
| 7002 | PRDX6 | 1.32 | 1.01 | 1.45 | -1.65 | -1.72 | -1.30 | **0.041** | 0.067 | **0.019** |  |  |  | -1.65 | -1.72 |  |
| 2010 | ARMCX1 | -1.56 | 1.23 | -1.32 | 1.23 | -1.61 | -3.19 | 0.156 | 0.127 | **0.037** |  | 1.23 |  |  | -1.61 |  |
| 9603 | FSCN1 | 2.20 | 2.07 | 1.31 | -1.26 | -1.32 | 1.43 | 0.110 | 0.500 | **0.025** | 2.20 | 2.07 | 1.31 | -1.26 |  | 1.43 |
| 7301 | HNRNPAB | -1.09 | -1.46 | -1.14 | 1.81 | 2.46 | 1.79 | 0.375 | 0.562 | **0.047** |  | -1.46 |  |  | 2.46 |  |
| 8007 | TPI1 | 2.32 | 1.23 | -1.14 | -1.45 | -1.02 | 1.25 | 0.426 | 0.242 | **0.002** | 2.32 |  |  | -1.45 |  |  |
| 6208 | HTRA2 | 1.33 | 1.01 | 1.59 | 1.95 | 1.56 | 1.96 | **0.026** | **0.040** | 0.764 | 1.31 | 1.31 | 1.31 | 1.83 | 1.83 | 1.83 |
| 1009 | JARID2 | -1.29 | -1.10 | -1.38 | -1.69 | -1.41 | -1.26 | **0.000** | **0.045** | 0.534 | -1.26 | -1.26 | -1.26 | -1.45 | -1.45 | -1.45 |
| 7504 | ALDH1A1 | -1.13 | 1.07 | 1.03 | 1.14 | 1.46 | 1.57 | **0.023** | 0.535 | 0.855 | -1.01 | -1.01 | -1.01 | 1.39 | 1.39 | 1.39 |
| 6502 | ANXA7 | -1.63 | -1.02 | -1.42 | -1.00 | 1.11 | -1.63 | **0.042** | 0.206 | 0.621 | -1.36 | -1.36 | -1.36 | -1.17 | -1.17 | -1.17 |
| 5205 | AUH | -1.23 | -1.14 | -1.28 | 1.32 | 1.14 | -1.04 | **0.027** | 0.832 | 0.677 | -1.22 | -1.22 | -1.22 | 1.14 | 1.14 | 1.14 |
| 4701 | CCT5 | 1.16 | 1.20 | 1.28 | 1.07 | 1.14 | 1.04 | **0.013** | 0.414 | 0.794 | 1.21 | 1.21 | 1.21 | 1.08 | 1.08 | 1.08 |
| 1008 | GLRX3 | -1.22 | -1.39 | -1.20 | -1.61 | -1.68 | -1.63 | **0.016** | 0.189 | 0.948 | -1.27 | -1.27 | -1.27 | -1.64 | -1.64 | -1.64 |
| 301 | HSPA2 | -2.10 | -1.56 | -1.33 | -1.14 | -1.13 | -1.61 | **0.022** | 0.178 | 0.305 | -1.66 | -1.66 | -1.66 | -1.29 | -1.29 | -1.29 |
| 6306 | PGK1 | -1.10 | 1.10 | 1.08 | -1.18 | 1.19 | -1.35 | **0.025** | 0.672 | 0.834 | 1.03 | 1.03 | 1.03 | -1.11 | -1.11 | -1.11 |
| 8901 | ALB | 1.54 | 1.35 | 1.42 | 1.38 | 1.69 | 1.28 | 0.777 | **0.024** | 0.410 | 1.46 | 1.52 |  | 1.46 | 1.52 |  |
| 7503 | CSTF1 | 1.19 | 1.05 | 1.44 | 1.42 | 1.40 | 1.73 | 0.510 | **0.033** | 0.822 |  |  | 1.59 |  |  | 1.59 |
| 3704 | HNRNPK | 1.10 | -1.08 | -1.01 | 1.80 | 1.29 | 1.53 | 0.116 | **0.008** | 0.070 | 1.45 |  |  | 1.45 |  |  |
| 3702 | HSPD1 | 1.23 | 1.06 | 1.42 | 2.45 | 2.66 | 3.30 | 0.687 | **0.003** | 0.123 |  |  | 2.36 |  |  | 2.36 |
| 5403 | KRT18 | -1.16 | -1.27 | -2.05 | -1.70 | -1.02 | -1.43 | 0.439 | **0.032** | 0.239 |  |  | -1.74 |  |  | -1.74 |
| 2601 | KRT7 | 1.55 | 2.17 | 2.08 | 1.61 | 1.69 | 1.43 | 0.930 | **0.018** | 0.589 |  | 1.93 |  |  | 1.93 |  |
| 3204 | PDHB | 1.16 | 1.18 | 1.51 | 1.26 | 1.29 | 1.38 | 0.439 | **0.008** | 0.649 |  |  | 1.44 |  |  | 1.44 |
| 7204 | PKM | 1.10 | 1.35 | -1.37 | -1.38 | 1.04 | -1.62 | 0.257 | **0.044** | 0.662 |  | 1.20 | -1.49 |  | 1.20 | -1.49 |
| 8301 | RBM4 | 1.77 | 1.44 | 1.91 | 1.23 | 1.44 | 1.73 | 0.053 | **0.002** | 0.413 |  |  | 1.82 |  |  | 1.82 |
| 1203 | SEC13 | -1.28 | -1.04 | 1.32 | 1.02 | 1.27 | 1.16 | 0.080 | **0.040** | 0.140 | -1.13 |  | 1.24 | -1.13 |  | 1.24 |
| 5706 | TCP1 | 1.46 | -1.10 | 1.60 | -1.15 | -1.60 | 1.01 | 0.134 | **0.034** | 0.375 |  | -1.35 | 1.30 |  | -1.35 | 1.30 |
| 1602 | TUBA1C | 1.27 | -1.72 | 2.46 | -1.32 | -2.51 | -1.29 | 0.296 | **0.019** | 0.117 |  | -2.11 | 1.59 |  | -2.11 | 1.59 |
| 2902 | *VCP | 1.17 | -1.13 | 1.44 | 1.06 | -1.17 | 1.58 | 0.091 | **0.005** | 0.730 |  |  | 1.51 |  |  | 1.51 |
| 2904 | *VCP | 1.22 | -1.35 | 1.17 | -1.21 | -1.53 | 1.20 | 0.083 | **0.049** | 0.491 |  | -1.44 | 1.19 |  | -1.44 | 1.19 |
| 107 | YWHAE | 1.64 | 1.12 | -1.36 | 1.14 | 1.08 | -1.38 | 0.206 | **0.039** | 0.728 | 1.39 |  | -1.37 | 1.39 |  | -1.37 |

**^§^** Spot volume intensity normalized to the control (n = 3).

**^†^** Significant change in protein expression identified by multiple comparison based on Holm-Sidak method (see Materials and Methods), which was used for pathway analysis, and the blank entries imply non-significant changes as compared to the control (i.e., fold-change = 1.0). Those protein spots with p-value < 0.05 (based on Two-way ANOVA) but did not pass Holm-Sidak test were excluded.

* Protein represented by multiple spots. The spot with the highest fold-change was used for bioinformatics.

Table S2. Primers used in RT-PCR to detect expression of genes in A549 cells.

| **Gene** | **Forward Primer** | **Reverse Primer** | **% Efficiency** |
| --- | --- | --- | --- |
| *ALDH1A1* | TGTTAGCTGATGCCGACTTG | CTGGATGCGGCTATACAACA | 98.32 |
| *ANXA5* | CAAGTTGAACAAGATGCTCAGG | TCTTCATCTGTCCCCCATTT | 95.57 |
| *ANXA7* | ACAGATGCCTTCTCAGTATC | GCTGACTAGGGTAAGTAGGTT | 97.84 |
| *ATM* | CTATGGAAATTAAGGTGGAC | AATTTACACCTCCTGCTAAG | 98.18 |
| *ACTB* | GCACCCAGCACAATGAAGA | CGATCCACACGGAGTACTTG | 93.19 |
| *BAX* | AGCTCTGAGCAGATCATGAAGA | GATCCTGGATGAAACCCTGA | 96.67 |
| *CASP1* | GGGGTACAGCGTAGATGTGAA | TGCTGTCAGAGGTCTTGTGC | 99.08 |
| *CASP3* | GCTATTGTGAGGCGGTTGTAG | CAGGGCTCGCTAACTCCTC | 95.70 |
| *CASP8* | TCCAAATGCAAACTGGATGA | TCTCCCAGGATGACCCTCTT | 96.83 |
| *CAT* | GCCATTGCCACAGGAAAG | CCTTGTGAGGCCAAACCTT | 97.09 |
| *CCL5* | CCTCATTGCTACTGCCCTCT | GGTGTGGTGTCCGAGGAATA | 98.67 |
| *CCNG1* | GCACAGAAGTGTGTAGAGTTAACAGA | AGCTCTTGCCAGAAGGTCAG | 92.12 |
| *CDKN1A* | CCAGCTGAGGTGTGAGCA | TGACATGGCGCCTCCT | 93.62 |
| *JUN* | CCCCATCGACATGGAGTC | CTCTCCAGCTTCCTTTTTCG | 97.81 |
| *CSF2* | TCTCAGAAATGTTTGACCTCCA | GCCCTTGAGCTTGGTGAG | 108.88 |
| *CSTF1* | CGGTAGATTGGGCAGGATT | CTGGTCCGGTTCTCTTGGTA | 106.13 |
| *CYP1A1* | CCAGGCTCCAAGAGTCCAC | AAGCATGATCAGTGTAGGGATCT | 106.29 |
| *CYP1B1* | ACGTACCGGCCACTATCACT | CTCGAGTCTGCACATCAGGA | 100.47 |
| *DDIT3* | TGCTTTTCCAGACTGATCCA | GACAGTGTCCCGAAGGAGAA | 95.15 |
| *DEK* | CCGAGAAAGAACCCGAAAT | CCTTCCACGATGAGACTCTTTT | 95.25 |
| *DNMT3A* | GACCCTCCAAAGGTTTACCC | CCAAGTCCTTCAGCACCAG | 96.94 |
| *DNMT3B* | TCCTATCGAAAAGCCATGTACC | TGGTCCTCCAATGAGTCTCC | 101.43 |
| *ECE1* | ACAGATGCCTGCTCAACAACT | GCCCAGGTTGTTTTCTGTGT | 92.13 |
| *EDNRA* | GCGCTCTTAGTGTTGACAGGT | GAATCCCAATTCCCTGAACA | 89.45 |
| *EGR1* | GGCCCTCAATACCAGCTACC | AAGCGTAAGGGCGTTCGT | 101.26 |
| *NOS3* | CGGAGAATGGAGAGAGATGG | CTCACGTCTATAATCGCAGCA | 97.79 |
| *EPHX1* | ACTGGCGGAATGAATTTGAC | CACGTGGATGAAGTGGATGT | 100.95 |
| *ERCC1* | GAAACCAGCGGACCTCCT | CACGGTGGTCAGACATTCAG | 97.53 |
| *ERCC3* | TGTCCTCATTCAGATCTCATCC | TTGTACTCTTCTGCAACCATCC | 92.11 |
| *EDN1* | GCTCGTCCCTGATGGATAAA | CTCTTGGACCTAGGGCTTCC | 92.14 |
| *EDN2* | GTGCCACCTTCTGCCTTC | CACGTCTGCAGGGGACTT | 91.43 |
| *EDN3* | CAAAGAAGAGGAAGGGAAGGTT | GGGGGCAGGTAGATGGAG | 100.98 |
| *FMO5* | ATTAGCCAAACAGCCAAGCA | ACACGATTCAGGATCCAAGC | 99.44 |
| *FSCN1* | GCCAACGAGAGGAACGTG | GGCACACTTTTTGGTGTCG | 98.14 |
| *GADD45A* | GGAGAGCAGAAGACCGAAAG | AGTGATCGTGCGCTGACTC | 100.08 |
| *GAPDH* | AGCCACATCGCTCAGACAC | GCCCAATACGACCAAATCC | 99.02 |
| *GPX1* | CAACCAGTTTGGGCATCAG | GTTCACCTCGCACTTCTCG | 98.77 |
| *GRSF1* | TGGATGATGTCTTTCTCATTCG | CTCACCGTTGCGGATTCT | 95.40 |
| *GSR* | TGCCAGCTTAGGAATAACCAG | CCTGCACCAACAATGACG | 96.34 |
| *GSTP1* | CCCTCATCTACACCAACTAT | AGCGAAGGAGATCTGG | 96.43 |
| *HMOX* | AGGGTGATAGAAGAGGCCAAG | CTGGTCCTTGGTGTCATGG | 99.19 |
| *HNRNPAB* | CTACGACTACTCGCCCTAT | TACTACCCTGACTGTAGTCGT | 96.61 |
| *HPRT1* | AAGATGGTCAAGGTCGCAAG | CCAACAAAGTCTGGCTTATATCC | 92.99 |
| *HSP90AB1* | GCTTGGAATCCACGAAGACT | TCTCCAGACTGGGAGGTATGA | 91.48 |
| *HSPA1A* | CCGGCCTACTTCAACGACT | GATGATCCGCAGCACGTT | 94.02 |
| *HSPA2* | AGGTGATCAACTGGCTCGAC | CGAGCTCTTTCTGCTTGTGTT | 99.42 |
| *HSPA5* | GATATTGGAGGTGGGCAAAC | TAACAACTGCATGGGTAACCTTC | 97.38 |
| *HSPA6* | CCGCCTATTTCAATGACTCG | ATTGATGATCCGCAACACG | 100.36 |
| *HSPA9* | AGGTGGGGAAGACTTTGACC | TCCTTCACAATGTGCCGTAG | 97.54 |
| *HSPB1* | TCCCTGGATGTCAACCACTT | GATGTAGCCATGCTCGTCCT | 96.02 |
| *HSPD1* | CCTGCACTCTGTCCCTCACT | GGTAACCGAAGCATTTCTGC | 94.88 |
| *HSPE1* | ACAGTAGTCGCTGTTGGATCG | AGAACTACTTTGGTGCCTCCAT | 95.81 |
| *HSPH1* | AGCCATGTTGTTGACTAAGCTG | TCTGTAAAGAAGGAGGGGACTG | 94.64 |
| *HTRA2* | ATTGGGGTGATGATGCTGAC | AGCTTGGTTCTCGAAGCTGT | 99.10 |
| *ICAM1* | GTGCAATCATGGTTCACTGC | GGTGTGGTGTTGTGAGCCTA | 96.20 |
| *IL1B* | CAGCCAATCTTCATTGCTCA | AGTCATCCTCATTGCCACTGT | 97.16 |
| *IL8* | GAGCACTCCATAAGGCACAAA | ATGGTTCCTTCCGGTGGT | 104.62 |
| *INA* | GCGAGGAGACACGTTTTAGC | GTGGGAGCAGGTAACTTGGA | 98.05 |
| *NOS2* | CCAGTACGTTTGGCAATGG | CCAAACACAGCGTACCTGAA | 98.79 |
| *KDM6B* | GGCACCAACATCGACTTGT | GTGGATGTTACCCGCATGA | 94.88 |
| *LTA* | AACCTGCTGCTCACCTCATT | TGCTCAAGGAGAAACCATCC | 93.31 |
| *MT1A* | GGTTCCTGCAAGTGCAAAG | CCTGGGCACACTTGCTACA | 97.83 |
| *MT2A* | TGCACCTCCTGCAAGAAA | CAGCAGCTGCACTTGTCC | 97.43 |
| *MYC* | AATGTCAAGAGGCGAACACA | TCCGTTTTAGCTCGTTCCTC | 95.19 |
| *NFAT5* | GGCACAATGAACCAACTGC | GCTGGTCCAGAGGTTAAAAGC | 92.84 |
| *NFKB1* | ATGCTCAGGAGCAGAAGTCC | GTCCACATGGGCATCACC | 99.29 |
| *OGG1* | CTCCACTCCTGCCCTGTG | CAGTGTGCAGGACTTTGCTC | 98.36 |
| *PDHB* | CAGGTGACAGTTCGTGATGC | TCCAAGCAGAAATACCTTCTCAT | 95.99 |
| *POR* | ACAACCTGGATGAGGAGT | TGGTGATGTCCAGGTAGT | 97.74 |
| *POU4F1* | CTCCCTGAGCACAAGTACCC | GGCGAAGAGGTTGCTCTG | 98.25 |
| *PRDX1* | GGTTGAACCCCAAGCTGATA | CAGCTGTGGCTTTGAAGTTG | 99.52 |
| *PRDX2* | GCCTTCCAGTACACAGACGAG | GTTGGGCTTAATCGTGTCACT | 95.79 |
| *PRDX6* | CCCAACTTTGAGGCCAATAC | GTCTCCCAGAAAGTCGTGGA | 96.15 |
| *PTGS1* | TGCGCCTGGTACTCACAGT | CGAGTGTAATAGCTCACGTTGG | 95.94 |
| *RPL32* | GAAGTTCCTGGTCCACAACG | AGCGATCTCGGCACAGTAAG | 96.49 |
| *SEC13* | CATTATCTGGAGAGAGGAAAACG | AGCACACCGAGTTCACTGAG | 94.07 |
| *SEMG1* | AAATGACAAGGTCGGCTCAG | GATCCACCTTTTTGTCCCATC | 94.49 |
| *SOD2* | AATCAGGATCCACTGCAAGG | TAAGCGTGCTCCCACACAT | 96.84 |
| *TCP1* | TTTTGAAGCTGCAATGTTGG | TGCAGACGTACGAGCCTTAG | 94.24 |
| *TIMP1* | GCTTCTGGCATCCTGTTGTT | ACTTGGCCCTGATGACGA | 91.78 |
| *TNFa* | CAGTCAGATCATCTTCTCG | GCTTGAGGGTTTGCTAC | 100.23 |
| *TNFSF10* | TTCACAGTGCTCCTGCAGTC | GCCACTTTTGGAGTACTTGTCC | 98.45 |
| *TP53* | AGGCCTTGGAACTCAAGGAT | GGTAGACTGACCCTTTTTGGAC | 100.66 |
| *TUBB4B* | CTGCTGCTGTTTGTCTACTTCC | CTGCAAGTGCACGATTTCC | 95.18 |
| *VHL* | CATCCACAGCTACCGAGGTC | CCGTCAACATTGAGAGATGG | 95.17 |
| *VIM* | CAAAGACAGGCTTTAGCGAGTT | GACAAGAGCGCCCCTAAGTT | 106.62 |
| *XRCC1* | AAAGAAGACCCCCAGCAAAC | TGGAGCTGGCAATTTAGGTC | 98.79 |
| *XRCC2* | ACCCATCTCTCTGCCTTTTG | ATTGACGCGGTCTATCCAGT | 95.92 |
| *YWHAZ* | AGACAGCACGCTAATAATGCAA | AATGAGGCAGACAAAAGTTGG | 99.49 |

Table S3. Two-way ANOVA results showing significant alteration of genes in A549 cells due to particle exposures (n = 3). The number below *Treatment* main effect (Trt), *Dose* main effect (Dose) or interaction between *Treatment and Dose* (T x D) corresponds to the p-value, where the bolded number emphasized p-value<0.05. The fold-change of gene indicated in blue (cut-off at ±1.10) was excluded from pathway analysis.

|  | ^§^**Cristobalite (μg/cm^2^)** | | | ^§^**Min-U-Sil 5 (μg/cm^2^)** | | | **Two-way ANOVA** | | | **^†^Cristobalite (μg/cm^2^)** | | | **^†^Min-U-Sil 5 (μg/cm^2^)** | | |
| --- | --- | --- | --- | --- | --- | --- | --- | --- | --- | --- | --- | --- | --- | --- | --- |
| **Gene** | **60** | **140** | **200** | **60** | **140** | **200** | **Trt** | **Dose** | **T x D** | **60** | **140** | **200** | **60** | **140** | **200** |
| DDIT3 | 1.10 | 1.19 | 1.44 | 1.86 | 2.12 | 2.28 | **0.000** | **0.000** | **0.014** |  |  |  | 1.86 | 2.12 | 2.28 |
| EDNRA | -1.00 | -1.02 | -1.00 | -1.64 | -1.93 | -2.29 | **0.000** | **0.004** | **0.005** |  |  |  | -1.64 | -1.93 | -2.29 |
| EPHX1 | 1.18 | -1.01 | -1.08 | -1.41 | -1.84 | -2.04 | **0.000** | **0.010** | **0.035** | 1.18 |  |  | -1.41 | -1.84 | -2.04 |
| EDN3 | -1.07 | -1.07 | -1.36 | -1.87 | -3.20 | -5.64 | **0.000** | **0.000** | **0.005** |  |  |  | -1.87 | -3.20 | -5.64 |
| FMO5 | -1.20 | -1.71 | -1.95 | -2.78 | -5.36 | -7.01 | **0.000** | **0.000** | **0.003** |  | -1.71 | -1.95 | -2.78 | -5.36 | -7.01 |
| ICAM1 | 1.11 | 1.54 | 2.58 | 2.32 | 3.26 | 6.50 | **0.005** | **0.000** | **0.034** | 1.11 | 1.54 | 2.58 | 2.32 | 3.26 | 6.50 |
| IL1B | 1.26 | -1.34 | -1.71 | -1.41 | -2.24 | -1.26 | **0.048** | **0.006** | **0.030** | 1.26 | -1.34 | -1.71 | -1.41 | -2.24 |  |
| MT1A | 1.11 | -1.04 | -1.09 | -1.29 | -1.60 | -1.54 | **0.000** | **0.005** | **0.046** | 1.11 |  |  | -1.29 | -1.60 | -1.54 |
| MYC | 1.11 | 1.30 | 1.26 | 1.47 | 1.60 | 1.64 | **0.000** | **0.000** | **0.022** |  | 1.30 | 1.26 | 1.47 | 1.60 | 1.64 |
| TNF | -1.24 | 3.06 | -1.60 | 4.80 | 8.55 | 15.40 | **0.000** | **0.010** | **0.024** | -1.24 |  | -1.60 | 4.80 | 8.55 | 15.40 |
| HSPA2 | 1.02 | 1.05 | 1.05 | -1.08 | -1.13 | -1.27 | **0.001** | 0.160 | **0.016** |  | 1.05 |  |  | -1.13 | -1.27 |
| NFAT5 | 1.04 | 1.26 | 1.17 | 1.29 | 1.09 | 1.10 | 0.893 | 0.076 | **0.040** |  |  |  | 1.29 |  |  |
| ALDH1A1 | -1.05 | -1.11 | -1.23 | -1.38 | -1.56 | -1.90 | **0.000** | **0.000** | 0.098 |  |  |  | -1.38 | -1.56 | -1.90 |
| CAT | -1.05 | -1.09 | -1.15 | -1.24 | -1.42 | -1.48 | **0.004** | **0.003** | 0.178 |  |  |  |  | -1.42 | -1.48 |
| DNMT3B | 1.01 | 1.00 | -1.06 | -1.10 | -1.31 | -1.41 | **0.004** | **0.015** | 0.106 |  |  |  | -1.10 | -1.31 | -1.41 |
| EGR1 | -1.65 | -2.70 | -4.48 | -2.71 | -5.82 | -8.46 | **0.036** | **0.000** | 0.257 | -1.65 | -2.70 | -4.48 | -2.71 | -5.82 | -8.46 |
| HSPA6 | 1.45 | 1.76 | 2.32 | 1.86 | 2.74 | 4.51 | **0.021** | **0.007** | 0.260 | 1.45 | 1.76 | 2.32 | 1.86 | 2.74 | 4.51 |
| IL8/CXCL8 | 1.24 | 1.46 | 2.30 | 2.23 | 3.09 | 4.96 | **0.001** | **0.000** | 0.062 | 1.24 | 1.46 | 2.30 | 2.23 | 3.09 | 4.96 |
| PDHB | 1.03 | -1.03 | -1.08 | -1.19 | -1.34 | -1.50 | **0.000** | **0.003** | 0.061 |  |  |  |  | -1.34 | -1.50 |
| PRDX2 | -1.10 | -1.21 | -1.26 | -1.39 | -1.59 | -1.60 | **0.002** | **0.005** | 0.582 |  | -1.21 | -1.26 |  | -1.59 | -1.60 |
| SEMG1 | 1.43 | 1.62 | 2.96 | 2.00 | 2.91 | 8.05 | **0.016** | **0.003** | 0.766 |  | 1.62 | 2.96 |  | 2.91 | 8.05 |
| TP53 | 1.18 | 1.00 | -1.21 | -1.39 | -1.75 | -1.90 | **0.008** | **0.028** | 0.161 |  |  |  |  |  | -1.90 |
| CCL5 | -1.19 | -1.04 | 1.27 | 2.00 | 2.83 | 3.20 | **0.010** | 0.068 | 0.187 | 1.01 | 1.01 | 1.01 | 2.68 | 2.68 | 2.68 |
| CSF2 | 2.11 | 4.80 | 2.66 | 11.57 | 10.71 | 35.02 | **0.049** | 0.233 | 0.262 | 3.19 | 3.19 | 3.19 | 19.10 | 19.10 | 19.10 |
| CYP1A1 | 1.08 | 1.02 | 1.13 | -1.22 | 1.10 | -1.19 | **0.028** | 0.734 | 0.204 | 1.08 | 1.08 | 1.08 | -1.10 | -1.10 | -1.10 |
| DNMT3A | -1.02 | 1.03 | -1.10 | -1.13 | -1.29 | -1.36 | **0.040** | 0.177 | 0.416 | -1.03 | -1.03 | -1.03 | -1.26 | -1.26 | -1.26 |
| ECE1 | -1.02 | 1.08 | 1.04 | -1.17 | -1.25 | -1.34 | **0.008** | 0.571 | 0.208 |  |  |  | -1.25 | -1.25 | -1.25 |
| ERCC3 | -1.06 | 1.01 | -1.13 | -1.13 | -1.29 | -1.25 | **0.015** | 0.090 | 0.246 |  |  |  | -1.22 | -1.22 | -1.22 |
| EDN1 | 1.04 | 1.06 | -1.00 | -1.07 | -1.24 | -1.25 | **0.028** | 0.352 | 0.237 |  |  |  | -1.19 | -1.19 | -1.19 |
| GADD45A | 1.20 | 1.12 | 1.06 | 1.17 | 1.28 | 1.39 | **0.021** | 0.141 | 0.272 |  |  |  | 1.28 | 1.28 | 1.28 |
| GSR | 1.02 | -1.01 | -1.10 | -1.09 | -1.20 | -1.34 | **0.047** | 0.052 | 0.494 |  |  |  | -1.21 | -1.21 | -1.21 |
| HSPA1A | 1.17 | 1.29 | 1.16 | 1.50 | 1.42 | 1.53 | **0.015** | 0.107 | 0.586 | 1.21 | 1.21 | 1.21 | 1.48 | 1.48 | 1.48 |
| OGG1 | 1.02 | 1.04 | 1.00 | -1.07 | -1.17 | -1.23 | **0.018** | 0.375 | 0.290 |  |  |  | -1.16 | -1.16 | -1.16 |
| PRDX1 | 1.01 | 1.07 | 1.01 | 1.13 | 1.17 | 1.17 | **0.028** | 0.071 | 0.338 |  |  |  | 1.16 | 1.16 | 1.16 |
| PTGS1 | -1.22 | -1.18 | -1.16 | -1.55 | -1.80 | -2.06 | **0.030** | 0.058 | 0.456 |  |  |  | -1.80 | -1.80 | -1.80 |
| CDKN1A | -1.26 | -1.21 | -1.35 | -1.20 | -1.33 | -1.27 | 0.223 | **0.026** | 0.827 |  |  | -1.31 |  |  | -1.31 |
| TNFSF10 | -1.46 | -2.00 | -2.12 | -1.39 | -1.91 | -2.02 | 0.850 | **0.000** | 0.990 | -1.43 | -1.96 | -2.07 | -1.43 | -1.96 | -2.07 |

^§^ Average fold-change of gene compared to the control (n = 3).

^†^ Significant change in gene expression identified by Holm-Sidak multiple comparisons, which was used for pathway analysis. Those genes with p-value < 0.05 (based on Two-way ANOVA) but did not pass Holm-Sidak test were excluded.

Table S4. Pearson Correlations indicating the cytotoxic effects that significantly associated (p < 0.05) with the dose of exposure in A549 cells. Correlation was conducted by correlating Log_2_(fold-effect) from each cytotoxicity assay against the doses expressed in mass and surface area (SA) metrics, where R corresponds to the correlation coefficient and p-value < 0.05 indicates a significant correlation. Two-way ANOVA results were also included in this table to show the significant cytotoxicity caused by CR and MI exposures. The highlighted numbers in blue and red pointed out p-value less than 0.05 based on Pearson correlation and two-way ANOVA analyses.

|  |  |  |  | **Pearson Correlation** | | | |
| --- | --- | --- | --- | --- | --- | --- | --- |
|  | **Two-Way ANOVA (p-value)** | | | **Mass (μg/cm^2^)** | | **SA (mm^2^/cm^2^)** | |
| **Cytotoxicity Assay** | **Trt** | **Dose** | **TxD** | **R** | **p-value** | **R** | **p-value** |
| LDH Release | 0.138 | **0.000** | 0.236 | 0.663 | **0.000** | 0.716 | **0.000** |
| BrdU Incorporation | 0.683 | **0.002** | 0.915 | -0.605 | **0.000** | -0.584 | **0.000** |
| Cellular ATP | 0.906 | **0.000** | 0.652 | -0.395 | **0.025** | -0.324 | 0.070 |
| Resazurin Reduction | 0.472 | 0.506 | 0.944 | 0.170 | 0.352 | 0.089 | 0.626 |

Table S5. The protein spots that were significantly correlated between their expressions and exposure doses based on Pearson correlation. Correlation was done by correlating Log_2_(fold-change) of each protein spot against the doses expressed in mass and surface area (SA) metrics as shown in Table 1, where R corresponds to the correlation coefficient and p-value < 0.05 indicates a significant correlation. The SSP number corresponds to the identifier number that PDQuest used to identify the spot based on its coordinate in the gel. #N/A indicates the protein spots whose identity is not available. Two-way ANOVA result was also included in this table to show the protein spots that were differentially expressed in A549 cells due to CR and MI exposures. The highlighted numbers in blue and red pointed out p-value less than 0.05 based on Pearson correlation and two-way ANOVA analyses.

|  |  |  |  |  | **Pearson Correlation** | | | |
| --- | --- | --- | --- | --- | --- | --- | --- | --- |
|  |  | **Two-Way ANOVA** | | | **Mass (μg/cm^2^)** | | **SA (mm^2^/cm^2^)** | |
| **SSP** | **Protein** | **Trt** | **Dose** | **TxD** | **R** | **p-value** | **R** | **p-value** |
| 3702 | HSPD1 | 0.687 | **0.003** | 0.123 | 0.511 | **0.011** | 0.725 | **0.000** |
| 4002 | HSPB1 | 0.629 | 0.055 | 0.493 | -0.537 | **0.007** | -0.616 | **0.001** |
| 6009 | NT5C | 0.059 | 0.118 | 0.138 | -0.362 | 0.082 | -0.613 | **0.001** |
| 1005 | PNMA6A | **0.042** | 0.086 | **0.043** | 0.314 | 0.135 | 0.603 | **0.002** |
| 3204 | PDHB | 0.439 | **0.008** | 0.649 | 0.654 | **0.001** | 0.597 | **0.002** |
| 7503 | CSTF1 | 0.510 | **0.033** | 0.822 | 0.504 | **0.012** | 0.583 | **0.003** |
| 2805 | HSPA9 | 0.360 | 0.158 | 0.313 | 0.361 | 0.083 | 0.574 | **0.003** |
| 5006 | NDUFV2 | 0.528 | 0.142 | 0.510 | 0.456 | **0.025** | 0.573 | **0.003** |
| 1401 | ACTB | 0.642 | 0.100 | 0.830 | 0.510 | **0.011** | 0.573 | **0.003** |
| 2603 | #N/A | 0.709 | 0.247 | 0.302 | 0.443 | **0.030** | 0.561 | **0.004** |
| 9403 | METTL18 | 0.568 | 0.106 | 0.394 | 0.424 | **0.039** | 0.559 | **0.005** |
| 802 | HSPA5 | 0.986 | 0.246 | 0.324 | 0.371 | 0.075 | 0.556 | **0.005** |
| 1711 | HSPA9 | 0.470 | 0.077 | 0.256 | 0.505 | **0.012** | 0.555 | **0.005** |
| 8301 | RBM4 | 0.053 | **0.002** | 0.413 | 0.658 | **0.000** | 0.552 | **0.005** |
| 1502 | #N/A | 0.410 | 0.186 | 0.828 | 0.469 | **0.021** | 0.551 | **0.005** |
| 4101 | PHB | 0.829 | 0.238 | 0.080 | 0.275 | 0.194 | 0.547 | **0.006** |
| 1108 | C1QTNF9B | 0.225 | 0.066 | 0.540 | 0.405 | **0.050** | 0.544 | **0.006** |
| 1004 | KRT81 | 0.446 | 0.228 | 0.414 | 0.346 | 0.097 | 0.543 | **0.006** |
| 6307 | #N/A | 0.303 | 0.219 | 0.564 | 0.406 | **0.049** | 0.536 | **0.007** |
| 3709 | #N/A | 0.634 | 0.095 | 0.870 | 0.450 | **0.028** | 0.536 | **0.007** |
| 1306 | ACTB | 0.787 | 0.204 | 0.693 | 0.362 | 0.082 | 0.534 | **0.007** |
| 5102 | PSME3 | 0.102 | 0.052 | 0.602 | -0.535 | **0.007** | -0.532 | **0.007** |
| 3007 | PHB | 0.383 | 0.104 | 0.917 | 0.480 | **0.017** | 0.531 | **0.008** |
| 7202 | MRPS22 | 0.444 | 0.181 | 0.145 | 0.333 | 0.112 | 0.527 | **0.008** |
| 5301 | XXYLT1 | 0.633 | 0.200 | 0.446 | 0.460 | **0.024** | 0.527 | **0.008** |
| 7508 | #N/A | 0.521 | 0.416 | 0.623 | 0.375 | 0.071 | 0.525 | **0.008** |
| 3816 | #N/A | 0.617 | 0.284 | 0.302 | 0.307 | 0.144 | 0.525 | **0.008** |
| 2010 | ARMCX1 | 0.156 | 0.127 | **0.037** | -0.387 | 0.062 | -0.519 | **0.009** |
| 7206 | BCO2 | 0.498 | 0.543 | 0.507 | 0.351 | 0.092 | 0.516 | **0.010** |
| 3705 | INA | 0.530 | **0.041** | 0.988 | 0.514 | **0.010** | 0.516 | **0.010** |
| 1508 | PSMC3 | 0.737 | 0.171 | 0.579 | 0.441 | **0.031** | 0.511 | **0.011** |
| 8203 | AKR1B1 | 0.947 | 0.304 | 0.607 | -0.324 | 0.122 | -0.509 | **0.011** |
| 6702 | BRCC3 | 0.921 | 0.109 | 0.554 | -0.370 | 0.075 | -0.509 | **0.011** |
| 1003 | EIF1AX | 0.282 | 0.145 | 0.077 | 0.272 | 0.198 | 0.508 | **0.011** |
| 4802 | HSPA9 | 0.078 | **0.045** | 0.198 | 0.420 | **0.041** | 0.506 | **0.012** |
| 4505 | #N/A | 0.692 | 0.136 | 0.683 | 0.358 | 0.086 | 0.502 | **0.012** |
| 1203 | SEC13 | 0.080 | **0.040** | 0.140 | 0.477 | **0.019** | 0.501 | **0.013** |
| 2702 | HSPD1 | 0.473 | 0.709 | 0.233 | 0.242 | 0.255 | 0.494 | **0.014** |
| 1006 | RBM8A | 0.249 | 0.088 | 0.990 | 0.532 | **0.007** | 0.494 | **0.014** |
| 6302 | PSMC5 | 0.237 | 0.235 | 0.766 | 0.389 | 0.060 | 0.489 | **0.015** |
| 8602 | #N/A | 0.951 | 0.172 | 0.065 | -0.235 | 0.269 | -0.485 | **0.016** |
| 7504 | ALDH1A1 | **0.023** | 0.535 | 0.855 | 0.347 | 0.096 | 0.484 | **0.017** |
| 6005 | PSMB3 | 0.423 | 0.097 | 0.134 | -0.274 | 0.195 | -0.483 | **0.017** |
| 6605 | PKM | 0.079 | 0.208 | 0.154 | -0.294 | 0.163 | -0.480 | **0.018** |
| 3001 | #N/A | 0.136 | 0.458 | 0.705 | 0.348 | 0.096 | 0.479 | **0.018** |
| 4207 | #N/A | 0.342 | 0.818 | 0.115 | 0.188 | 0.380 | 0.474 | **0.019** |
| 804 | #N/A | 0.065 | 0.221 | 0.638 | 0.433 | **0.035** | 0.473 | **0.019** |
| 7607 | ALDH1A1 | 0.057 | **0.047** | 0.163 | -0.391 | 0.059 | -0.468 | **0.021** |
| 3802 | HSPA1A | 0.391 | 0.274 | 0.758 | 0.374 | 0.072 | 0.465 | **0.022** |
| 4303 | MSN | 0.511 | 0.177 | 0.466 | -0.327 | 0.119 | -0.460 | **0.024** |
| 7805 | ANKLE2 | 0.441 | 0.287 | 0.153 | -0.239 | 0.262 | -0.459 | **0.024** |
| 4004 | PRDX4 | 0.835 | 0.569 | 0.449 | 0.243 | 0.253 | 0.458 | **0.024** |
| 9805 | #N/A | 0.872 | 0.230 | 0.207 | 0.343 | 0.100 | 0.456 | **0.025** |
| 2303 | ACTB | 0.383 | 0.578 | 0.568 | 0.346 | 0.098 | 0.456 | **0.025** |
| 4406 | #N/A | 0.883 | 0.063 | 0.976 | -0.461 | **0.023** | -0.455 | **0.026** |
| 8606 | PKM | 0.645 | 0.107 | 0.372 | -0.422 | **0.040** | -0.454 | **0.026** |
| 6704 | CSTF2 | 0.606 | 0.437 | 0.608 | 0.342 | 0.102 | 0.449 | **0.028** |
| 5705 | PDIA3 | 0.234 | 0.166 | 0.465 | 0.515 | **0.010** | 0.448 | **0.028** |
| 6504 | #N/A | 0.269 | 0.083 | 0.153 | -0.250 | 0.240 | -0.448 | **0.028** |
| 6208 | HTRA2 | **0.026** | **0.040** | 0.764 | 0.398 | 0.054 | 0.446 | **0.029** |
| 6703 | #N/A | 0.693 | 0.205 | 0.338 | 0.489 | **0.015** | 0.445 | **0.030** |
| 9502 | DIS3L | 0.781 | 0.146 | 0.103 | -0.386 | 0.062 | -0.443 | **0.030** |
| 6203 | PKM | 0.164 | 0.120 | 0.833 | -0.350 | 0.094 | -0.441 | **0.031** |
| 3701 | HNRNPK | 0.387 | 0.290 | 0.398 | -0.411 | **0.046** | -0.439 | **0.032** |
| 1503 | PSMC4 | 0.331 | 0.195 | 0.513 | 0.402 | 0.052 | 0.435 | **0.033** |
| 8501 | EEF2 | 0.058 | 0.078 | 0.643 | 0.273 | 0.197 | 0.435 | **0.034** |
| 6402 | ACTB | 0.588 | 0.055 | 0.832 | 0.389 | 0.060 | 0.432 | **0.035** |
| 8307 | FAH | 0.220 | 0.185 | 0.339 | 0.219 | 0.303 | 0.429 | **0.037** |
| 2704 | HSPD1 | 0.542 | 0.135 | 0.577 | 0.460 | **0.024** | 0.427 | **0.037** |
| 9202 | HNRNPDL | 0.733 | 0.483 | 0.577 | 0.259 | 0.221 | 0.426 | **0.038** |
| 5107 | #N/A | 0.840 | 0.098 | 0.111 | 0.414 | **0.044** | 0.424 | **0.039** |
| 1804 | HSPA5 | 0.723 | 0.217 | 0.326 | 0.339 | 0.105 | 0.422 | **0.040** |
| 6604 | CCT2 | 0.449 | 0.205 | 0.445 | 0.333 | 0.112 | 0.420 | **0.041** |
| 3506 | KRT8 | 0.161 | 0.144 | 0.233 | -0.358 | 0.086 | -0.419 | **0.041** |
| 2915 | HSP90AA1 | 0.460 | 0.233 | 0.775 | -0.331 | 0.114 | -0.418 | **0.042** |
| 4702 | PDIA3 | 0.278 | 0.297 | 0.285 | -0.260 | 0.219 | -0.417 | **0.042** |
| 8105 | #N/A | 0.504 | 0.456 | 0.101 | -0.273 | 0.196 | -0.412 | **0.045** |
| 7808 | LMNA | 0.564 | 0.755 | 0.301 | -0.194 | 0.362 | -0.411 | **0.046** |
| 3707 | HNRNPK | 0.237 | 0.106 | 0.416 | -0.337 | 0.107 | -0.409 | **0.047** |
| 8901 | ALB | 0.777 | **0.024** | 0.410 | 0.430 | **0.036** | 0.407 | **0.048** |
| 2005 | ANXA1 | 0.307 | 0.102 | 0.932 | -0.404 | 0.050 | -0.405 | **0.049** |
| 2601 | KRT7 | 0.930 | **0.018** | 0.589 | 0.570 | **0.004** | 0.401 | 0.052 |
| 3101 | OFD1 | 0.369 | 0.057 | 0.859 | -0.518 | **0.010** | -0.379 | 0.068 |
| 5105 | PSMB7 | 0.874 | 0.125 | 0.546 | 0.513 | **0.010** | 0.400 | 0.053 |
| 8801 | #N/A | **0.019** | **0.038** | 0.195 | 0.510 | **0.011** | 0.351 | 0.093 |
| 7106 | #N/A | 0.085 | 0.122 | 0.613 | 0.467 | **0.021** | 0.215 | 0.313 |
| 5403 | KRT18 | 0.439 | **0.032** | 0.239 | -0.466 | **0.022** | -0.366 | 0.079 |
| 6401 | TUBB4B | 0.768 | **0.036** | 0.478 | -0.464 | **0.022** | -0.397 | 0.055 |
| 2405 | #N/A | 0.429 | 0.181 | 0.556 | -0.455 | **0.025** | -0.402 | 0.052 |
| 104 | IPO4 | 0.506 | 0.082 | 0.765 | 0.446 | **0.029** | 0.348 | 0.096 |
| 7203 | RGS1 | 0.731 | 0.102 | 0.410 | 0.435 | **0.034** | 0.236 | 0.268 |
| 1507 | #N/A | 0.592 | 0.151 | 0.771 | -0.430 | **0.036** | -0.325 | 0.121 |
| 7609 | GART | 0.831 | 0.134 | 0.874 | 0.427 | **0.037** | 0.341 | 0.103 |
| 7010 | BAG2 | 0.339 | 0.239 | 0.460 | 0.422 | **0.040** | 0.205 | 0.336 |
| 3109 | #N/A | 0.764 | 0.243 | 0.822 | -0.418 | **0.042** | -0.307 | 0.145 |
| 5602 | #N/A | 0.310 | 0.134 | 0.099 | 0.406 | **0.049** | 0.306 | 0.146 |

Table S6. Pearson correlation conducted to identify significant responses of genes in A549 cell to the exposure dose regardless of the type of silica particle. Correlation was done by correlating Log_2_(fold-change) of each gene against the doses expressed in mass and surface area (SA) as shown in Table 1, where R corresponds to the correlation co-efficient and p-value < 0.05 indicates a significant correlation. Two-way ANOVA result was also included in this table to show the genes that were differentially expressed in A549 cells due to CR and MI exposures. The highlighted numbers in blue and red pointed out p-value less than 0.05 based on Pearson correlation and two-way ANOVA analyses.

|  |  |  |  | **Pearson Correlation** | | | |
| --- | --- | --- | --- | --- | --- | --- | --- |
|  | **Two-Way ANOVA** | | | **Mass (μg/cm^2^)** | | **SA (mm^2^/cm^2^)** | |
| **Gene** | **Trt** | **Dose** | **TxD** | **R** | **p-value** | **R** | **p-value** |
| *EGR1* | **0.036** | **0.000** | 0.257 | -0.907 | **0.000** | -0.953 | **0.000** |
| *FMO5* | **0.000** | **0.000** | **0.003** | -0.714 | **0.000** | -0.910 | **0.000** |
| *IL8/CXCL8* | **0.001** | **0.000** | 0.062 | 0.782 | **0.000** | 0.901 | **0.000** |
| *ICAM1* | **0.005** | **0.000** | 0.073 | 0.778 | **0.000** | 0.896 | **0.000** |
| *HSPA6* | **0.021** | **0.007** | 0.260 | 0.743 | **0.000** | 0.861 | **0.000** |
| *EDN3* | **0.000** | **0.000** | **0.005** | -0.613 | **0.001** | -0.858 | **0.000** |
| *ALDH1A1* | **0.000** | **0.000** | 0.098 | -0.640 | **0.001** | -0.839 | **0.000** |
| *MYC* | **0.000** | **0.000** | **0.022** | 0.702 | **0.000** | 0.832 | **0.000** |
| *CAT* | **0.004** | **0.003** | 0.178 | -0.621 | **0.001** | -0.795 | **0.000** |
| *TNFSF10* | 0.850 | **0.000** | 0.990 | -0.865 | **0.000** | -0.794 | **0.000** |
| *SEMG1* | 0.064 | **0.020** | 0.235 | 0.652 | **0.001** | 0.789 | **0.000** |
| *DDIT3* | **0.000** | **0.000** | **0.014** | 0.592 | **0.002** | 0.787 | **0.000** |
| *PDHB* | **0.000** | **0.003** | 0.061 | -0.561 | **0.004** | -0.786 | **0.000** |
| *EPHX1* | **0.000** | **0.010** | **0.035** | -0.520 | **0.009** | -0.757 | **0.000** |
| *TP53* | **0.008** | **0.028** | 0.161 | -0.565 | **0.004** | -0.746 | **0.000** |
| *DNMT3B* | **0.004** | **0.015** | 0.106 | -0.535 | **0.007** | -0.741 | **0.000** |
| *CSF2* | **0.049** | 0.233 | 0.262 | 0.540 | **0.006** | 0.734 | **0.000** |
| *EDNRA* | **0.000** | **0.004** | **0.005** | -0.459 | **0.024** | -0.732 | **0.000** |
| *MT1A* | **0.000** | **0.005** | **0.046** | -0.501 | **0.013** | -0.717 | **0.000** |
| *PRDX2* | **0.002** | **0.005** | 0.582 | -0.541 | **0.006** | -0.708 | **0.000** |
| *CCL5* | **0.010** | 0.068 | 0.187 | 0.507 | **0.011** | 0.685 | **0.000** |
| *GSR* | **0.047** | 0.052 | 0.494 | -0.536 | **0.007** | -0.683 | **0.000** |
| *EDN2* | 0.088 | 0.067 | 0.496 | 0.571 | **0.004** | 0.652 | **0.001** |
| *PTGS1* | **0.030** | 0.058 | 0.456 | -0.475 | **0.019** | -0.635 | **0.001** |
| *INA* | 0.155 | **0.033** | 0.828 | 0.565 | **0.004** | 0.627 | **0.001** |
| *TNF* | **0.015** | 0.124 | 0.122 | 0.387 | 0.062 | 0.624 | **0.001** |
| *VHL* | 0.069 | 0.065 | 0.801 | -0.525 | **0.008** | -0.613 | **0.001** |
| *CCNG1* | 0.415 | 0.064 | 0.646 | -0.551 | **0.005** | -0.612 | **0.001** |
| *PRDX6* | 0.080 | 0.106 | 0.512 | -0.494 | **0.014** | -0.609 | **0.002** |
| *ERCC3* | **0.015** | 0.090 | 0.246 | -0.444 | **0.030** | -0.608 | **0.002** |
| *HSPA2* | **0.001** | 0.160 | **0.016** | -0.328 | 0.118 | -0.605 | **0.002** |
| *DNMT3A* | **0.040** | 0.177 | 0.416 | -0.431 | **0.036** | -0.599 | **0.002** |
| *YWHAZ* | 0.198 | 0.063 | 0.574 | 0.510 | **0.011** | 0.582 | **0.003** |
| *CDKN1A* | 0.223 | **0.026** | 0.827 | -0.545 | **0.006** | -0.581 | **0.003** |
| *PRDX1* | **0.028** | 0.071 | 0.338 | 0.412 | **0.046** | 0.575 | **0.003** |
| *GADD45A* | **0.021** | 0.141 | 0.272 | 0.376 | 0.070 | 0.567 | **0.004** |
| *OGG1* | **0.018** | 0.375 | 0.290 | -0.349 | 0.094 | -0.560 | **0.004** |
| *NOS3* | 0.230 | 0.238 | 0.765 | 0.488 | **0.015** | 0.558 | **0.005** |
| *JMJD* | **0.001** | 0.107 | 0.837 | 0.402 | 0.052 | 0.556 | **0.005** |
| *IL1B* | 0.050 | **0.037** | 0.100 | -0.498 | **0.013** | -0.551 | **0.005** |
| *EDN1* | **0.028** | 0.352 | 0.237 | -0.351 | 0.093 | -0.550 | **0.005** |
| *HSPA1A* | **0.015** | 0.107 | 0.586 | 0.378 | 0.068 | 0.532 | **0.007** |
| *SOD2* | 0.079 | 0.097 | 0.730 | 0.420 | **0.041** | 0.531 | **0.008** |
| *HSPA5* | 0.586 | 0.102 | 0.964 | 0.530 | **0.008** | 0.501 | **0.013** |
| *GAPDH* | 0.208 | 0.277 | 0.744 | -0.412 | **0.045** | -0.485 | **0.016** |
| *CYP1B1* | 0.083 | 0.498 | 0.199 | -0.294 | 0.164 | -0.471 | **0.020** |
| *ECE1* | **0.008** | 0.571 | 0.208 | -0.212 | 0.319 | -0.463 | **0.023** |
| *FSCN1* | 0.711 | 0.087 | 0.818 | 0.527 | **0.008** | 0.448 | **0.028** |
| *CASP1* | 0.707 | **0.016** | 0.202 | -0.560 | **0.004** | -0.446 | **0.029** |
| *XRCC2* | 0.406 | 0.336 | 0.750 | 0.404 | 0.050 | 0.434 | **0.034** |
| *NOS2* | 0.240 | 0.244 | 0.465 | 0.372 | 0.073 | 0.434 | **0.034** |
| *POR* | 0.805 | 0.184 | 0.868 | 0.458 | **0.024** | 0.431 | **0.035** |
